# Regulation of plant Ni uptake by soil-borne microorganisms occurs independently of their Ni-solubilizing capabilities

**DOI:** 10.1093/ismejo/wraf265

**Published:** 2025-12-01

**Authors:** Agnieszka Domka, Maciej Gustab, Roman J Jędrzejczyk, Rafał Ważny, Alice Tognacchini, Markus Puschenreiter, Paweł Łabaj, Agata Muszyńska, Weronika Kosowicz, Kinga Jarosz, Piotr Rozpądek

**Affiliations:** Polish Academy of Sciences, W. Szafer Institute of Botany, Lubicz 46, Kraków 31-512, Poland; Malopolska Centre of Biotechnology, Jagiellonian University in Kraków, Gronostajowa 7a, Kraków 30-387, Poland; Malopolska Centre of Biotechnology, Jagiellonian University in Kraków, Gronostajowa 7a, Kraków 30-387, Poland; Doctoral School of Exact and Natural Sciences, Jagiellonian University in Kraków, Łojasiewicza 11, Kraków 30-387, Poland; Malopolska Centre of Biotechnology, Jagiellonian University in Kraków, Gronostajowa 7a, Kraków 30-387, Poland; Malopolska Centre of Biotechnology, Jagiellonian University in Kraków, Gronostajowa 7a, Kraków 30-387, Poland; Vienna, Department of Forest and Soil Sciences, Institute of Soil Research, University of Natural Resources and Life Sciences, Konrad-Lorenz Straße 24, Tulln 3430, Austria; Vienna, Department of Forest and Soil Sciences, Institute of Soil Research, University of Natural Resources and Life Sciences, Konrad-Lorenz Straße 24, Tulln 3430, Austria; Malopolska Centre of Biotechnology, Jagiellonian University in Kraków, Gronostajowa 7a, Kraków 30-387, Poland; Malopolska Centre of Biotechnology, Jagiellonian University in Kraków, Gronostajowa 7a, Kraków 30-387, Poland; Malopolska Centre of Biotechnology, Jagiellonian University in Kraków, Gronostajowa 7a, Kraków 30-387, Poland; Doctoral School of Exact and Natural Sciences, Jagiellonian University in Kraków, Łojasiewicza 11, Kraków 30-387, Poland; Faculty of Geography and Geology, Institute of Geological Sciences, Jagiellonian University, Gronostajowa 3a, Kraków 30-387, Poland; Malopolska Centre of Biotechnology, Jagiellonian University in Kraków, Gronostajowa 7a, Kraków 30-387, Poland

**Keywords:** abiotic stress adaptation, microbiota, metal hyperaccumulation

## Abstract

Plant-associated microbial communities play a vital role in host adaptation to environmental stress, yet their contributions to plant nickel (Ni) tolerance strategies remain unclear. It is not understood whether the same microbial community elicits similar responses across different plant species or regulates stress adaptation in a host-specific manner. Although microorganisms influence plant responses to metal toxicity by altering metal bioavailability in the rhizosphere, their potential to optimize plant metal uptake is less explored. In this study, we evaluated whether synthetic microbial communities enhance Ni uptake in two species with contrasting metal strategies: the hyperaccumulator *Odontarrhena chalcidica* and the Ni-excluding *Arabidopsis arenosa*. We hypothesized that soil microorganisms support plant metal adaptation by improving physiological function rather than altering soil metal availability. Our results show that *O. chalcidica* reached its full hyperaccumulating potential only when co-cultivated with a soil-derived microbial community, regardless of the microorganisms’ ability to mobilize Ni or promote plant growth. Microorganisms that enhanced Ni uptake had no effect on soil Ni availability. Microbial community analysis revealed species-specific microbiota assembly, with *O. chalcidica* being more responsive yet more selective. Serpentine-soil microbiota enhanced Ni uptake in *O. chalcidica* by upregulating iron-transporter genes, confirming reliance on Fe-transport pathways for Ni acquisition. In contrast, the same inoculum induced Zn-transporters and NRT2.1/NRT2.2 in *A. arenosa*, reflecting strategy of cation partitioning and nutrient-transport fine-tuning under Ni stress. These findings refine criteria for selecting microorganisms in phytoremediation and highlight that the functional impact of plant-associated microorganisms on metal handling outweigh their effects on metal solubility in soil.

## Graphical Abstract

**Graphical Abstract:**
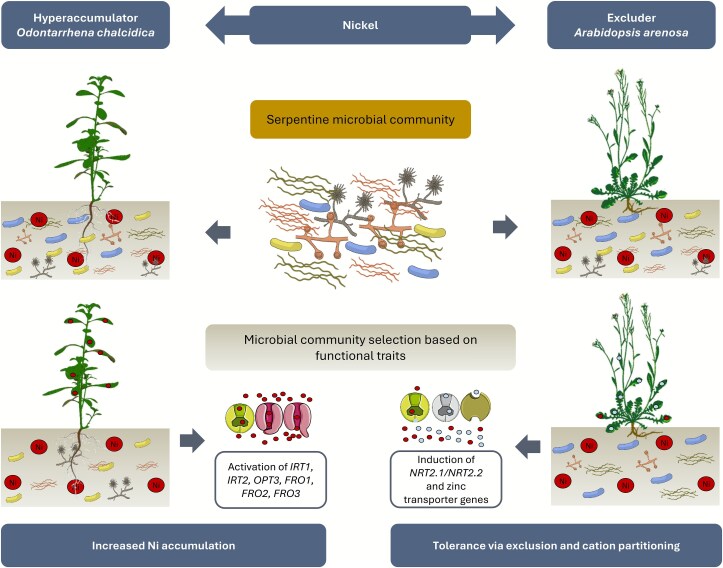

## Introduction

Natural selection, as a fundamental mechanism of evolution, operates at various levels, ranging from genes to entire ecosystems. Since 1990, when Lynn Margulis introduced the “holobiont” concept into the field of life sciences [1], it has become widely accepted that living organisms cannot be understood in isolation but as complex associations of hosts and microorganisms, collectively termed the holobiont. The holobiont, along with collective genetic information of all genomes within, known as the hologenome, constitutes an evolutionary unit subject to the pressure of natural selection [2–4]. The adaptation of the holobiont to the environment and its overall fitness results from the intricate interactions between the symbionts and the host, as well as among the symbiotic microorganisms themselves [5, 6]. Microorganisms that adapt more rapidly to environmental dynamics are considered essential in the holobiont’s ability to respond to external cues and adapt accordingly.

Plants, as sessile organisms, continually confront environmental stressors, among which heavy metals pose significant challenges to their growth and development [7]. Whereas essential in trace amounts, excess heavy metals can be toxic, prompting plants to evolve distinct strategies for coping: metal detoxification and exclusion. Hyperaccumulators efficiently take up, detoxify, and sequester metals, whereas metal excluders employ mechanisms to limit metal uptake. The majority of identified plant metal hyperaccumulators possess the ability to accumulate high quantities of Ni [8, 9]. However, the role of microorganisms in mediating these strategies remains underexplored [10]. Microorganisms are known to affect metals’ mobility and availability to the plant, by releasing organic acids and other metal chelators, and influencing redox potential in the soil [11, 12]. Certain microorganisms can specifically mobilize nickel (Ni); for instance, *Psychrobacter* spp. and bacteria from the rhizosphere of *Alyssum murale* have been shown to increase Ni solubility, likely via siderophore production [11, 13]. Siderophores, primarily facilitating iron uptake, can also form stable complexes with Ni, enhancing its availability to plants [14, 15] (reviewed by [16]). Additionally, microbial activities involved in the solubilization of inorganic phosphates, accompanied by the reduction of soil pH, can enhance the availability of metals [17]. Together, these activities and plant growth promotion properties indirectly influence plants’ adaptation to metal-rich soils.

Serpentine soils, covering ~1% of the Earth’s land surface, are characterized by elevated concentrations of metals such as Ni, Fe, Mn, and Co, alongside deficiencies in essential nutrients such as nitrogen, phosphorus, and potassium [18, 19]. Moreover, their shallow profile and low water-holding capacity render them susceptible to drought and erosion, presenting a challenging environment for plant growth [20]. In this study, we aimed to elucidate the role of soil microorganisms in plant adaptation to toxic metals, with a particular focus on how plant microbiota affects host’s metal tolerance strategies. We hypothesized that microorganisms naturally co-occurring with plants in toxic metal-containing soils enhance the plant’s mechanisms for coping with metal stress, independent of their plant growth-promoting properties or their impact on metal bioavailability in the soil. This microbial influence is essential for optimizing the plant’s potential for metal hyperaccumulation or exclusion. To further characterize the role of microbial communities in shaping plant metal-accumulation strategies, we investigated the impact of microorganisms derived from serpentine soil and other metal-rich soil on metal accumulation in two plant species with differing metal tolerance strategies: the Ni hyperaccumulator *Odontharrhena chalcidica* and the Ni excluder *Arabidopsis arenosa*. The latter is also known for its adaptation to serpentine soil, as evidenced by genomic data showing strong selection of genes involved in ion homeostasis in serpentine populations, making it a suitable model plant for the study of serpentine adaptation [21, 22].

## Materials and methods

### Seeds and soil collection

Seeds of *O. chalcidica* (Janka) Španiel, Al-Shehbaz, D.A.German & Marhold and serpentine soil were collected in Bernstein (47°24′44′′N, 16°15′3′′E), located in the eastern Austrian province of Burgenland. *A. arenosa* seeds were collected from post mining waste “Bolesław” (50°17′32′′N, 19°29′79′′E), located in the Southern Poland. Garden soil (ARO, Makro, PL) was purchased locally in Poland. Both the garden soil and the serpentine were sieved through a 2 mm diameter sieve mesh. Serpentine was dried at a room temperature before use. Chemical soil analyses were conducted as detailed in the Supplementary methods text. For the culture of plants, both types of soil were mixed in a ratio 1:1 (v/v). Garden soil was added to prevent growth retardation observed in pure serpentine under laboratory conditions. Part of the soil mixture was sterilized utilizing the autoclave (121°C, 223 kPa, 20 min) a day before the plant culture was set.

### Chemical analysis of the soil

Soil chemical properties, including water content, pH_(H₂O, CaCl₂)_, water holding capacity, organic matter, Kjeldahl nitrogen, total and labile phosphorus, and metal concentrations (FAAS/GFAAS), were determined as described in the Supplementary methods.

### Plant growth promoting properties of bacteria and fungi

 Plant growth–promoting traits of bacterial and fungal isolates, including phosphate solubilization, siderophore production, and Ni mobilization were assessed. Experimental procedures followed established protocols with modifications, as detailed in the Supplementary methods.

### Isolation and identification of microorganisms from serpentine soil

Microorganisms were isolated from serpentine soil and purified on selective media. Taxonomic identification was based on morphological traits and sequencing of ITS-LSU rRNA (fungi) or 16S rRNA genes (bacteria), with detailed protocols provided in the Supplementary methods.

### Plant culture

#### Pot culture

Seeds of *O. chalcidica* and *A. arenosa* were surface sterilized by rinsing them in 4% NaOCl for 4 min, then next in 96% EtOH for 2 min, and in 70% EtOH for 4 min. Next, they were rinsed three times in sterile water. Sterilized seeds were sown into garden soil mixed with sand (1:1) and stratified for 48 h, and next transferred into a growth chamber (Biogenet, PL) with a 16 h photoperiod under 100 μmol · m^−2^ · s^−1^ of light intensity, 21/17°C day/night temperature and 50% humidity for three weeks. After this time, plants were transferred into pots filled with unsterile or sterile serpentine mixed with sterile garden soil (one plant per pot, *N =* 5) and cultured in a growth chamber. Plants were inoculated twice with (i) synthetic microbial community showing plant growth-promoting properties (SynCom); (ii) microorganisms isolated from serpentine soil; (iii) serpentine soil extract, first on the day of transferring them into serpentine mixed with garden soil, and then seven days later. After four weeks of culture, the plants were collected, rinsed with 10 mM EDTA and deionized water, and residual water was removed with a paper towel. The fresh weight of plant shoots and roots was measured. Plants were dried at 80°C for 24 h, and the dry weight of their shoots and roots was measured.

#### *In vitro* culture

*O. chalcidica* and *A. arenosa* seeds were surface sterilized and sown into sterile ¼ Murashige-Skoog (MS) (Duchefa Biochemie, NL) medium with 0.8% sucrose in a petri dish and placed in the dark (4°C). After 48 h, the seeds were transferred to a growth chamber (Panasonic MLR-352H-PE, Korea) with a 16 h photoperiod under 100 μmol · m-2 · s^−1^ of light intensity, 21/17°C day/night temperature and 50% humidity for 10 days. After this time plants were transferred into Strullu-Romand medium (MSR) (Duchefa Biochemie, NL) containing 500 μM Ni (NiSO_4_·6H_2_O) (Chempur, PL) and 100 μM Mn (MnCl_2_·4H_2_O) (Chempur, PL). Seedlings were inoculated with serpentine soil extract by placing a drop of extract on the surface of the root with an inoculation loop. To exclude the influence of soil extract compounds other than microorganisms, plants were also inoculated with autoclaved serpentine soil extract. Plants that were not inoculated served as a control. After seven days, the plants were harvested, weighed, rinsed in 10 mM EDTA and deionized water and used for further analysis.

### Serpentine extract preparation

The serpentine soil extract was prepared by adding 1 g of unsterile serpentine soil to 30 ml of sterile 0.9% NaCl solution in a 100 ml flask. The flasks were shaken for 48 h (140 rpm, 26°C). After this period, half of the flasks containing the serpentine extract were sterilized by autoclaving (121°C, 20 min). To prepare the solution for watering plants in pot cultures, 90 ml of the either sterile or unsterile extracts were separately added to 0.4 L of sterile 0.9% NaCl solution (to reach optical density at 600 nm (OD_600_) of 0.5).

### Isolation and identification of microorganisms from serpentine soil

To isolate microorganisms from serpentine soil, ~1 g of soil sample was used. Isolated fungi and bacteria were identified by sequencing specific genetic regions. For details see the Supplementary method text. Fungal and bacterial sequences were submitted to NCBI (www.ncbi.nlm.nih.gov) and listed in Supplementary Table 1. Isolated bacterial and yeast strains were stocked in 25% glycerol and stored at −80°C. Pure fungal strains were preserved on slanted potato dextrose agar (PDA) medium in tubes at 4°C.

### Preparation of synthetic community inocula

Microbial inoculum was prepared from the composition of bacteria and fungi cultures. For each experiment, microbial inocula were prepared from fresh starter cultures derived from the original stocks. Bacterial starter cultures were grown in liquid Luria Broth (LB) two days before inoculum preparation, and fungal starter cultures were grown on PDA one week prior to ensure all strains, including slow-growing fungi, were actively growing. Microorganisms from these starter cultures were then used to inoculate the cultures ultimately used for the experimental inocula: solid LB cultures for bacteria and PDA cultures for fungi. Bacterial cultures of each strain were harvested by gently scraping the agar surface into 10 ml of sterile 0.9% (w/v) NaCl and adjusted to an optical density at 600 nm (OD_600_) of 0.5. From each OD-adjusted suspension, 1 ml was transferred into a collective reservoir of sterile 0.9% NaCl. Fungal inocula were prepared by aseptically excising a single 5 mm-diameter agar plug per strain with a sterile cork borer and adding each plug to the same reservoir. Finally, the combined bacterial and fungal suspensions were brought to a total volume of 1 L with sterile 0.9% NaCl. The solution with the microorganisms added was used as an inoculum to water the plants. Methods for microbial plant growth promoting properties were described in the Supplementary method text.

### Ni and Mn availability in the soil

To assess the effect of sterilization on the bioavailable Ni concentration in soil, 10 g of air-dried sterile and unsterile soil (serpentine and serpentine mixed with garden soil in a 1:1 ratio) were weighed and added to 25 ml of 1 M ammonium acetate solution. The mixture was then shaken for 2 h at 140 rpm and 20°C. After shaking, the solution was filtered through filter paper and diluted to 50 ml with deionized water. The samples were analyzed for Ni and Mn concentrations (For details see Supplementary methods).

### Metal concentration

Plants cultured as previously described were harvested, rinsed in 10 mM EDTA and deionized water, and dried at 80°C for 24 h. For a single sample, ~50 mg of root or 100 mg of shoot samples were weighed to analytical accuracy. For plant cultured *in vitro,* all five plants from petri dish were weighed. The plant material was prepared as previously described [23]. Nickel was measured using graphite furnace atomic absorption spectrometry (GF-AAS) with an autosampler (Thermo Scientific, iC3000) and ICP-MS (NexION 2000, Perkin Elmer, USA). The external standard calibration method was applied using AAS standard solutions (Sigma Aldrich). The international standard ERM-CD281 for validation was used.

### Ni staining and confocal microscopy

Nickel staining was performed with the use of Newport Green DCF Diacetate according to the manufacturer’s instructions. Stained roots were analyzed with a Zeiss Axio Imager 2 inverted confocal microscope (Zeiss, DE).

### Diffusive gradient in thin films

Soil metal availability was measured post-harvest using Chelex-100 resin DGT (Diffusive Gradients in Thin Films) gels [24]. Approximately 3 grams of each frozen soil sample, sieved to 2 mm, were analyzed due to sample limitations, following standard methods [25, 26]. Ten samples per treatment (NS, SE-, SE+) were saturated to 100% water-holding capacity and equilibrated for 24 h before DGT deployment. Each DGT sampler consisted of a 0.4 mm Chelex-100 resin gel disc, a 0.8 mm polyacrylamide diffusive gel disc, and a 0.45 μm membrane filter. Retrieved gels were rinsed, then eluted in 10 ml of 1 M HNO₃ for 24 h. Metal concentrations in the eluates were analyzed via ICP-MS (NexION 2000, Perkin Elmer, USA), and DGT-labile metal concentrations were calculated following the previously described method [25].

### SEM–EDX analysis

Fresh leaves of *O. chalcidica* from *in vitro* cultures were analyzed for microstructure and elemental composition using SEM-EDS. Imaging and mapping parameters are provided in the Supplementary methods.

### High-throughput sequencing and data processing

DNA was extracted from *O. chalcidica* and *A. arenosa* roots, leaves, seeds and from the serpentine:garden soil mixture as previously described [27, 28]. DNA was then purified with a MagMAX Microbiome Ultra Nucleic Acid Isolation Kit (Thermo Fisher Scientific) according to the producer instructions. DNA libraries were prepared for bacteria (16S V5-V7 rRNA gene, 799f AACMGGATTAGATACCCKG [29], 1193r ACGTCATCCCCACCTTCC [30]) and fungi (ITS1 rRNA gene, ITS1F CTTGGTCATTTAGAGGAAGTAA [31], ITS2 GCTGCGTTCTTCATCGATGC [32]). Double-indexed amplicons were sequenced on the NovaSeq 6000 System (Illumina) (2 × 250 bp) by Novogene (UK). The sequencing depth was 100, 000 reads per sample. NGS data were processed using QIIME2 software [33]. In the first step, adapter sequences and low-quality sequences were removed. Then, chimaera were removed, and paired sequences were joined and clustered with the DADA2 algorithm [34] to obtain initial amplicon sequence variants (ASVs). Species annotation was performed with UNITE database (version no 10.0; [35]) for fungi and SILVA database (version no 138) for bacteria [36].

ASVs were filtered for very low abundance and only ASVs with a relative abundance of at least 0.01% and a frequency of at least 50% samples in one treatment were used in further analysis.

The total number of V5-V7 16S rRNA genes reads reached 2 533 646, with an average number of 60 324 reads per sample. The quality control passed 1 932 629 reads, with an average 46 014 reads per sample. The rarefaction depth (determined based on the depth of the sample with the fewest reads) was 2 248, and Shannon Index values were similar at 10 000 (Supplementary Fig. 9) In total, 654 bacterial ASVs were identified.

The total number of ITS1 rRNA reads reached 2 775 974, with an average number of reads per sample of 66 094. The quality control passed 2 550 998 reads with an average 60 738 reads per sample. The rarefaction depth was 39 466 reads. In total, 633 fungal ASVs were identified.

The observed ASVs (the richness per sample of the number of ASVs that passed the abundance test) were used to estimate alpha diversity. Bray–Curtis dissimilarity index were calculated for beta diversity to evaluate the complexity of the community composition and compare the differences between groups. Non-metric multidimensional scaling (NMDS) was performed (Bray–Curtis dissimilarity index) to visualize the differences of samples in complex multi-dimensional data. Statistical significance of the differences between study sites shown at NMDS plot was tested with an ANOSIM test. Significantly differed taxa were determined by DESeq package in R.

### RNA sequencing and data processing

Total RNA was isolated from roots of *O. chalcidica* and *A. arenosa* cultured *in vitro* for 7 days on MSR medium supplemented with 500 μM Ni and 100 μM Mn (*N =* 5, 8–10 roots per sample), using the MagMAX Plant RNA Isolation Kit (Thermo Fisher Scientific) with on-column DNase I treatment (Sigma-Aldrich). Library preparation and sequencing (NovaSeq X Plus System) were performed by Novogene (UK): mRNA was enriched with poly-T magnetic beads, fragmented, and reverse-transcribed into double-stranded cDNA. Libraries underwent end repair, A-tailing, adapter ligation, size selection, PCR amplification, and purification. Quality and quantity were assessed by Qubit, qPCR, and Bioanalyzer.

For *A. arenosa*, reads were pseudo-aligned to the *A. thaliana* TAIR10 transcriptome using Kallisto v0.48.0, and transcript abundances were collapsed to gene level. Differential expression was determined with DESeq2 (BY-adjusted *P* < .05), and GO enrichment was tested via the parent–child Fisher method in topGO (*P* < .05).

The *O. chalcidica* transcriptome was de novo assembled with rnaSPAdes v4.2.0 (297 714 transcripts), filtered for ≥1 TPM in ≥1 sample, and clustered with CD-HIT v4.8.1 to 231 669 nonredundant transcripts. BUSCO assessed completeness (94.7%). Annotation was performed with Trinotate v4.0.2; only *Viridiplantae* hits were retained. Differential expression and GO analyses followed the *A. arenosa* pipeline. Orthology mapping and detailed GO interpretation are described in the Supplementary methods.

### Functional analysis of plant and soil microbiota

Functional profiling of bacterial communities was performed using PICRUSt2, and fungal guilds were predicted with FUNGuild; detailed methodological descriptions are provided in the Supplementary methods.

### Statistical analysis

Statistical analysis was carried out using Statistica ver. 13.0 (StatSoft). The Shapiro–Wilk test was applied to assess data normality, whereas Levene’s test was used to check the homogeneity of variances. For comparing two groups, t-tests and Dunnett’s test were employed, and for multiple group comparisons, one-way ANOVA followed by Tukey’s Honest Significant Difference (HSD) *post hoc* test was performed. Detailed *P* values for statistical tests can be found in Supplementary Table 9.

## Results

### Microbial plant growth promotion and nickel mobilization potential

**Figure 1:**
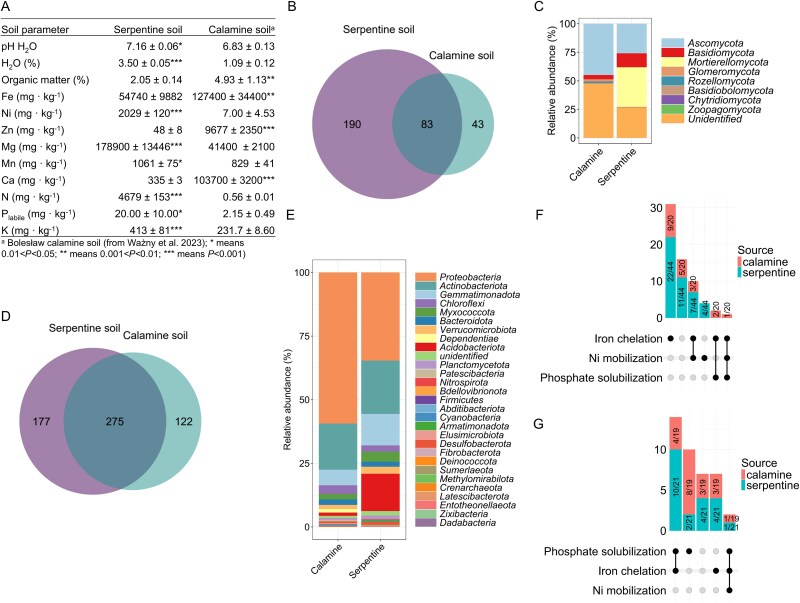
Serpentine and calamine soils differ in physicochemical properties and microbial community composition, but harbor functionally similar microorganisms: (A) physicochemical properties of serpentine soil and calamine soil from Bolesław heap. (B, D) Venn diagram visualizing the number of fungal (B) and bacterial (D) ASVs present in serpentine and calamine soils (*N =* 6). (C, E) structure of fungal (C) and bacterial (E) phyla identified in serpentine and calamine soils (*N =* 6). (F, G) upset plot illustrating the functional properties—phosphorus solubilization, iron chelation, and nickel mobilization—of bacterial (F) and fungal (G) strains isolated from serpentine and calamine soils.

Soil is one of the richest reservoirs of microorganisms and serves as a key source of the plant associated microbiota. We hypothesized that the microbial communities inhabiting different metal-rich soils, ultramafic soil from Berstein, Austria and calamine soil from Bolesław, Poland (Fig. 1A), would differ significantly in composition. However, we expected their functional potential for metal solubilization to be similar. First, we analyzed the microbial community structure of the soils by sequencing the ITS1 region and the V5–V7 regions of 16S rRNA genes (Supplementary methods, experimental design). The results revealed that serpentine soil shared ~30% of fungal taxa with calamine waste (Fig. 1B). *Ascomycota* dominated calamine soil (45.0%) but were less abundant in serpentine (26.0%). In contrast, *Mortierellomycota* was prominent in serpentine (34.6%) but absent from calamine. *Basidiomycota* were more abundant in serpentine (12.2%) than in calamine (3.7%). *Rozellomycota* was detected only in calamine (1.6%), whereas minor phyla such as *Glomeromycota* and *Chytridiomycota* were present at low levels in both soils. Unclassified sequences comprised a substantial portion in both environments (47.7% in Calamine, 26.1% in Serpentine) (Fig. 1C). Analysis of bacterial communities revealed a higher level of taxonomic overlap, with ~61% of bacterial taxa shared between the two soil types (Fig. 1D). *Proteobacteria* dominated both communities, accounting for nearly 60% of sequences in calamine soil and 34% in serpentine soil. The relative abundances of other major phyla were broadly similar between the two environments, with the exception of *Acidobacteriota*, which were significantly more abundant in serpentine soil (~15%) compared to calamine soil (~1%) (Fig. 1E). NMDS ordination based on Bray–Curtis dissimilarity and ANOSIM confirmed significant differences in microbial composition between both type of soils (Supplementary Fig. 1A and B). A more detailed description is provided in Supplementary Fig. 1 and in the plant microbiota analysis section of the results.

To investigate the functional potential of the microbial communities, we isolated bacteria and fungi from serpentine soil and assessed the ability of individual isolated strains to (i) mobilize Ni, (ii) produce siderophores, and (iii) solubilize phosphorus from insoluble inorganic phosphates (for molecular identification of microorganisms isolated from serpentine see Supplementary Table 1). In addition to testing all available isolates from serpentine soil, we also included 39 previously isolated strains from calamine soil (for molecular identification see Supplementary Table 2), many of which exhibit plant growth-promoting traits. The functional profiles of bacterial and fungal isolates from calamine and serpentine sources were analyzed separately (Supplementary Tables 3 and 4). For bacteria (Fig. 1F), isolates from both environments showed similar patterns in phosphate solubilization, iron chelation, and nickel mobilization. Iron chelation was the most common trait, whereas phosphate solubilization and nickel mobilization occurred less frequently but consistently across both populations. Similarly, fungal isolates from calamine and serpentine soils exhibited comparable functional capabilities, with phosphate solubilization being relatively frequent and iron chelation varying among strains. Nickel mobilization was less common but present in both fungal communities (Fig. 1G). Overall, these results indicate that microbial populations from calamine and serpentine soils share similar functional potentials despite differences in taxonomic composition.

### Nickel accumulation in plants is shaped by synthetic microbial community composition

A similar proportion of microorganisms from both soil types were capable of solubilizing Ni, confirming that this trait is common among metal-adapted microorganisms, regardless of their origin. Based on this, we hypothesized that microbial inoculation would optimize plant metal uptake independently of the microorganisms’ Ni-solubilization potential. Specifically, we expected that the inocula would stimulate Ni take up in *Odontarrhena chalcidica* (hyperaccumulator) and reduce it in *A. arenosa* (metal excluder). To test this, we performed a series of experiments using the serpentine soil microbial community applied to two plant species in the form of: native soil, soil extract and SynCom I2 composed from all microorganisms isolated from the soil (Supplementary methods, Experimental design). We compared the impact of serpentine microorganisms on plant Ni uptake and biomass production with that of the microbial community isolated from the “Bolesław” mine dump (SynCom I1). The I1 and I2 SynComs included all culturable microorganisms from calamine and serpentine soils, as described in the previous section. For a detailed description of the inocula composition, please refer to Supplementary Table 1, 2, 3 and 4.

**Figure 2:**
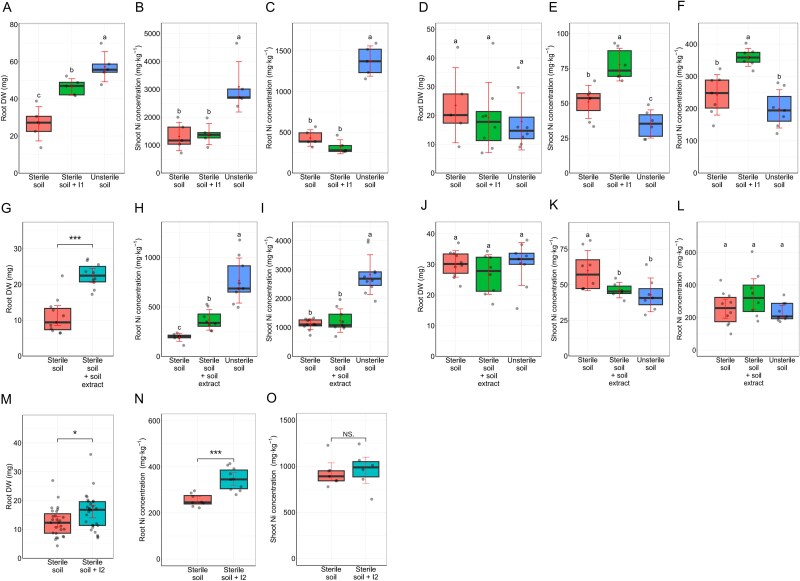
Impact of microbiota on *Odontarrhena chalcidica* and *A. arenosa* growth and Ni accumulation: (A) dry weight of *O. chalcidica* roots grown in sterilized serpentine soil (sterile soil), inoculated with inoculum with microorganisms isolated from calamine mine dump (sterile soil + **I1**) and grown in unsterile serpentine soil (unsterile soil) (*N =* 5). (B, C) Ni concentration in shoots (B) and roots (C) of *O. chalcidica* grown under the same treatments (*N =* 5). (D) Dry weight of *A. arenosa* roots grown in sterile soil, sterile soil + **I1** and unsterile soil (*N =* 5). (E, F) Ni concentration in shoots (E) and roots (F) of *A. arenosa* grown under the same treatments (*N =* 6–8). (G) Dry weight of *O. chalcidica* roots grown in sterilized serpentine soil (sterile soil) and inoculated with serpentine soil extract (sterile soil + soil extract) (*N =* 10–11). (H, I) Ni concentration in roots (H) and shoots (I) of *O. chalcidica* grown in sterile soil, sterile soil + **soil extract** and in unsterile soil (*N =* 8–10). (J) Dry weight of *A. arenosa* roots grown in sterile soil, sterile soil + **soil extract** and in unsterile soil (*N =* 8–10). (K, L) Ni concentration in shoots (K) and roots (L) of *A. arenosa* grown under the same treatments (*N =* 6–10). (M) Dry weight of *O. chalcidica* roots grown in sterile soil and inoculated with microorganisms isolated from serpentine soil (sterile soil + **I2**) (*N =* 9). (N, O) Ni concentration in roots (N) and shoots (O) of *O. chalcidica* grown under the same treatments (*N =* 10). Boxplots represent the median and interquartile range with standard deviation indicated in red. Stars indicate statistically significant differences (t-test, * - *P* ≤ .05, ** - *P* ≤ .01, *** - *P* ≤ .001) for pairwise comparisons and letters above bars denote significant differences between groups, as assessed by one-way ANOVA with Tukey’s post hoc test for all multiple comparisons (*P* ≤ .05). In all experiments, one plant was grown per pot.

In the first experiment we compared biomass production and Ni accumulation in *O. chalcidica* and *A. arenosa* under three conditions: **(i)** sterilized serpentine soil, **(ii)** serpentine soil inoculated with a synthetic microbial community derived from metal-rich calamine mining waste (SynCom **I1**), and **(iii)** unsterilized serpentine soil. The unsterilized soil served as a reservoir of serpentine soil microorganisms. After two months in growth chambers, we measured plant dry weight (DW). *O. chalcidica* roots produced significantly more biomass in unsterilized soil (an increase of over 100%) and in SynCom I1-inoculated soil (20% more) compared to the sterilized control (Fig. 2A). Neither treatment affected shoot biomass of *O. chalcidic*a (Supplementary Fig. 2A) nor root (Fig. 2D) nor shoot (Supplementary Fig. 2B) dry weight of *A. arenosa*.

We measured metal and other element concentration *in planta* with ICP-MS (Inductively Coupled Plasma Mass Spectrometry). Application of the calamine inoculum (I1) to *O. chalcidica* did not affect Ni uptake. In sterilized soil, plants accumulated 1310.6 ± 508.9 and 429.7 ± 100.9 mg · kg ^−1^ of Ni in the shoots and roots respectively (Fig. 2B and C). In addition to Ni, the accumulation of Mn, P, Na, K and Al levels were also altered by soil sterilization, but not inoculation (Supplementary Table 5, Supplementary Fig. 3C and D). These findings were further confirmed in an independent experiment using FAAS (Flame Atomic Absorption Spectroscopy; Supplementary Fig. 3A and B).

In the shoots of *A. arenosa*, the lowest Ni levels were observed in plants grown in unsterile serpentine (35.2 ± 10.1 mg·kg^−1^) which was significantly lower compared to uninoculated sterilized soil (51.0 ± 11.8 mg·kg^−1^). In contrast, inoculation of sterilized serpentine soil with I1 community significantly increased shoot Ni concentrations compared to other treatments, reaching 77.8 ± 11.7 (Fig. 2E).

*A. arenosa* roots accumulated comparable Ni levels whether grown in unsterilized serpentine (199.1 ± 59.4 mg·kg^−1^) or in sterilized soil without inoculum (242.7 ± 62.8 mg·kg^−1^). Only plants treated with calamine-derived microorganisms (I1) accumulated significantly more Ni in their roots (359.4 ± 27.8 mg·kg^−1^) (Fig. 2F).

The experiment demonstrated that depletion of the native soil microbiota led to a significant reduction in nickel accumulation by *Odontarrhena chalcidica* and increase in Ni take up by *A. arenosa*. We could not rule out the possibility that this effect was caused, at least in part, by soil sterilization itself. Indeed, it is well established that sterilization alters element availability regardless of the method used [37, 38], with manganese (Mn) availability being particularly affected by physical sterilization techniques [39]. In our study, Mn accumulation was elevated in *O. chalcidica* grown in sterilized soil (Supplementary Fig. 3C and D). Bioavailability measurements confirmed that sterilization did not affect Fe, Ca, K, or Mg levels, but significantly increased the availability of both Ni and Mn, thereby reducing the Ni:Mn (available) ratio from 0.1 to 0.02 (Supplementary Table 6). Ni uptake by *A. arenosa* was not inhibited by soil sterilization, suggesting that its metal uptake mechanisms differ from those of *O. chalcidica* and may be less sensitive to elevated Mn concentrations. To further investigate the role of serpentine microbiota in facilitating Ni uptake, we conducted a follow-up experiment in which sterilized serpentine soil was inoculated with: (iv) a synthetic community derived from serpentine soil (SynCom **I2**), and (v) a microbial suspension prepared as a water extract of the same soil to simplify application and provide a broad representation of the native microbial community (Supplementary methods, Experimental design). The root dry weight (DW) of *O. chalcidica* increased markedly in both treatments, reaching 16.6 ± 6.3 mg in the SynCom I2 treatment and 22.5 ± 3.3 mg in the soil extract treatment, compared to 12.6 ± 5 mg and 11.0 ± 4.9 mg in the respective uninoculated control (Fig. 2G and M), mirroring the response observed in unsterilized soil. Shoot biomass also increased with SynCom I2 but was unchanged with the extract (Supplementary Fig. 4A and B). In contrast, *A. arenosa* biomass production remained not affected by inoculation (Fig. 2J, Supplementary Fig. 4C). Whereas *O. chalcidica* exhibited improved growth following both treatments, Ni accumulation did not fully return to the levels observed in plants grown in nonsterilized serpentine soil. *O. chalcidica* plants treated with the serpentine-derived synthetic microbial community (SynCom I2) accumulated 35% more Ni in their roots compared to uninoculated controls (346.5 vs. 254.8 mg·kg^−1^), whereas no significant differences were observed in shoot Ni concentrations (Fig. 2N, O). A similar pattern was observed in plants grown in soil treated with the serpentine microbial extract. In this case, root Ni concentrations reached 353.1 ± 101.5 mg·kg^−1^ in inoculated plants, compared to 253.9 ± 116.2 mg·kg^−1^ in the controls and 767 ± 227.4 mg·kg^−1^in roots from unsterile soil (Fig. 2H). No significant differences were observed in shoot Ni concentrations between extract treated plants compared and controls, whereas shoot Ni concentrations in plants grown in unsterile soil were the highest, reaching 2827.3 ± 686.4 mg·kg^−1^ (Fig. 2I), confirming that inoculation enhanced Ni uptake into roots. Photosystem II activity measurements further indicated improved *O. chalcidica* performance with serpentine microbiota or soil extract, reflected in higher YII and ETRII (Supplementary Fig. 5A and B). Because Ni accumulation in *O. chalcidica* appeared unaffected by the method of microbiota application, we next examined the response of *A. arenosa*, which was co-cultivated in sterilized serpentine soil inoculated with a microbial extract. As in the initial experiment, *A. arenosa* grown in unsterilized soil accumulated significantly less Ni in shoots compared to those in sterilized soil (35.2 vs. 51.8 mg·kg^−1^; Fig. 2K). Also, inoculation with the microbial extract significantly decreased Ni uptake in *A. arenosa* shoots (Fig. 2K), whereas root accumulation remained unchanged regardless of sterilization or inoculation (Fig. 2L).

### Microorganisms activate Ni uptake independently of metal availability in the soil

**Figure 3:**
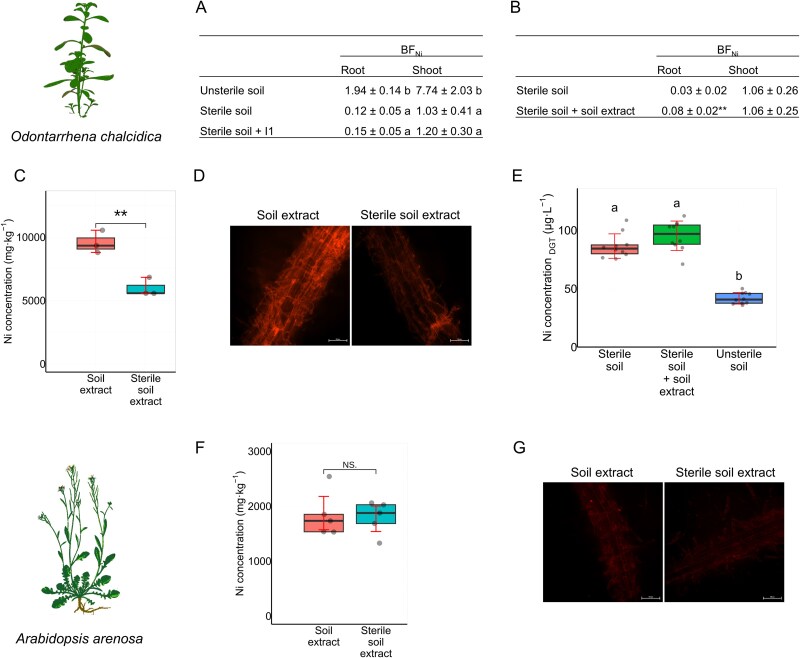
Microorganisms activate Ni uptake independently of metal availability in the soil: (A, B) Ni bioconcentration factor (BF) calculated for roots and shoots of *O. chalcidica* plants grown in sterile soil, sterile soil + I1, and unsterile soil (A) and in sterile soil and sterile soil + soil extract (B). (C) Ni concentration in whole *O. chalcidica* plants cultured *in vitro*.**For one sample one plate with 7–8 plants was harvested (*N =* 3). (D) Ni visualization in roots of *O. chalcidica.* Plants were cultured *in vitro* in medium containing 500 μM Ni and 100 μM Mn for 7 days. Half of the plants served as a control group, treated with sterile soil extract and remaining plants were treated with unsterilized serpentine soil extract. For each sample, three plates of plants were harvested, with 7–8 plants per plate, and stained with Newport green DCF diacetate (*N =* 3). (E) Microbial influence on the concentration of DGT-mobile Ni in serpentine soil: concentration of Ni in sterilized serpentine soil (sterile soil), sterilized serpentine soil supplemented with extract from serpentine soil (sterile soil + soil extract) and in unsterile serpentine soil (unsterile soil) (*N =* 10). (F) Ni concentration in whole *A. arenosa* plants cultured *in vitro* (*N =* 3). (G) Visualization of Ni distribution within the root system of *A. arenosa* (*N =* 3). Boxplots represent the median and interquartile range with standard deviation indicated in red. Stars indicate statistically significant differences (t-test, * - *P* ≤ .05, ** - *P* ≤ .01, *** - *P* ≤ .001) for pairwise comparisons and letters above bars denote significant differences between groups, as assessed by one-way ANOVA with Tukey’s post hoc test for all multiple comparisons (*P* ≤ .05).

To evaluate the *O. chalcidica’s* ability to access Ni from the soil Ni pool in the various treatments, the bioconcentration factor (BF), the ratio of metal concentration in the plant to the soil, was calculated. This approach allowed an evaluation of plant Ni uptake independently of Ni and other metal availability in the soil. The BF for Ni indicated hyperaccumulation in unsterile soil: 1.94 ± 0.14 for roots, 7.74 ± 2.03 for shoots, whereas sterilized soil drastically reduced BF values to 0.12 ± 0.05 and 1.03 ± 0.41 for the roots and shoots respectively. Inoculation with calamine microorganisms (I1) had a minimal effect on the BF (0.15 ± 0.05 for the roots and 1.20 ± 0.30 for the shoots; Fig. 3A). In another experiment, supplementation of the soil with the serpentine extract increased BF, calculated for roots, by close to 3-fold compared to uninoculated plants (Fig. 3B). For Mn, BF was low across all treatments but was slightly higher in plants grown in unsterile soil (Supplementary Fig. 3E).

To further demonstrate the critical role of microorganisms in enhancing plant uptake of nickel (Ni) and to eliminate the effects of sterilization on soil properties, an *in vitro* experiment was conducted using Strullu-Romand medium with nearly 100% Ni availability. *O. chalcidica* plants were cultured in a medium containing 500 μM Ni and 100 μM Mn, concentrations selected to reflect serpentine soil levels and optimized for *in vitro* culture so as not to be lethal to the plants thereby maintaining a consistent Ni:Mn ratio. Half of the plants were inoculated with serpentine soil extract, whereas the rest were inoculated with autoclaved extract as a control. Plants treated with the soil extract showed 30% higher Ni concentrations (9461 vs. 7268 mg·kg^−1^) compared to control (Fig. 3C). This was confirmed by Newport Green DCF Diacetate staining, revealing the highest fluorescence signal in the roots of plants treated with serpentine soil solution (Fig. 3D, Supplementary Fig. 6A). No significant differences in Mn concentration were observed between treated and control plants (Supplementary Fig. 6B). Additionally, SEM–EDX analysis was used to examine the presence of Ni in the leaf epidermis (Supplementary Fig. 6D and E). Diffusive gradient in thin films (DGT) technique was applied to evaluate the bioavailability of metals in pot cultures (Supplementary Figs 7A–D). The results show that the concentration of bioavailable Ni was significantly lower in unsterilized soil compared to sterilized soil (41.7 ± 4.9 μg·L^−1^ and 86.5 ± 10.4 μg·L^−1^ respectively). Supplementation with microorganisms (in soil extract) had no effect on Ni bioavailability (Fig. 3E). A similar trend was observed for Mn and Cu, whereas Fe and Zn were unaffected by either sterilization or extract inoculation (Supplementary Figs 7A–D).

For *A. arenosa* cultured *in vitro* no significant differences in Ni accumulation were found between treatments (Fig. 3F). DCF staining of roots confirmed no major differences among the groups (Fig. 3G, Supplementary Fig. 6C).

### *Odontarrhena chalcidica* and *A. arenosa* recruit distinct microbial communities from serpentine soil

To investigate the composition of the microbiota of both plant species, we performed high-throughput sequencing of V5-V7 16S rRNA genes and ITS1 amplicons from soil, as well as from the shoots, roots and seeds of *A. arenosa* and *O. chalcidica* cultured in ultramafic soil.

**Figure 4:**
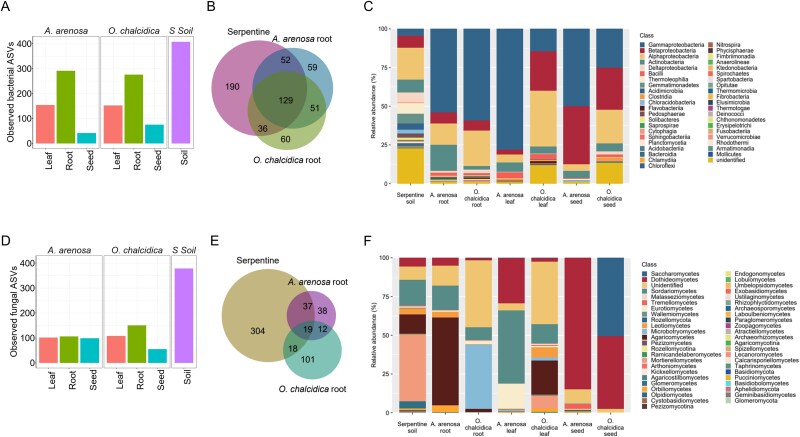
Comparative analysis of microbial diversity in *Arabidopsis arenosa* and *Odontarrhena chalcidica*: Number of amplicon sequence variants for bacteria (A) and fungi (D) inhabiting *A. arenosa* (Aa) and *O. chalcidica* (Oc) leaves, roots, seeds and serpentine soil. (B, D) Venn diagram visualizing the number of bacterial (B) and fungal (D) ASVs present in plant roots and serpentine soil (*N =* 6). (C, F) Structure of bacterial (C) and fungal (F) classes identified in serpentine soil and plant roots, leaves and seeds (*N =* 6).

Bacterial alpha diversity was highest in soil (407 ASVs), followed by roots, leaves, and seeds. In *O. chalcidica*, we identified 276 ASVs in roots, 152 in leaves, and 75 in seeds; *A. arenosa* harbored 291, 154, and 41 ASVs in the respective compartments (Fig. 4A). In *A. arenosa* roots, 52% of bacterial ASVs were shared with soil and 62% with *O. chalcidica* roots. Unique ASVs accounted for 20% and 22% of the root microbiomes of *A. arenosa* and *O. chalcidica*, respectively (Fig. 4B).

Soil bacterial communities were dominated by unidentified phyla (23%), *Alphaproteobacteria* (21%), and *Actinobacteria* (8%). In plant roots, *Gammaproteobacteria* were most abundant, comprising 54% (*A. arenosa*) and 59% (*O. chalcidica*) of the identified taxa. Other dominant classes included *Alphaproteobacteria*, *Actinobacteria*, and *Betaproteobacteria*. *A. arenosa* shoots were strongly dominated by *Gammaproteobacteria* (78%), whereas *O. chalcidica* exhibited higher proportions of *Alphaproteobacteria* (36%) and *Betaproteobacteria* (25%). Similar patterns were observed in seeds, with species-specific differences in the relative abundance of the dominant phyla (Fig. 4C, Supplementary Table 7, Supplementary Figs 8A–C).

Fungal diversity was also highest in soil (378 ASVs), with markedly lower richness in plant compartments (Fig. 4D). Community structure differed significantly between plant organs and between species. 53% (*A. arenosa*) and only 29% (*O. chalcidica*) of root-associated fungal ASVs overlapped with soil; 21% were shared between the two plant species. A high proportion of fungal ASVs were unique to each compartment, especially in roots (67% in *O. chalcidica*, 36% in *A. arenosa*) and soil (80%) (Fig. 4E).

Soil fungal communities were dominated by *Mortierellomycetes* (43%), *Sordariomycetes* (17%), and *Agaricomycetes* (12%). In roots, *A. arenosa* was enriched in *Agaricomycetes* (56%), whereas *O. chalcidica* was dominated by *Microbotryomycetes* (41%). Leaf-associated fungal communities were more variable and lacked a single dominant class. In seeds, *A. arenosa* was primarily colonized by *Dothideomycetes* (85%), whereas *O. chalcidica* had co-dominance of *Saccharomycetes* (51%) and *Dothideomycetes* (47%) (Fig. 4F, Supplementary Table 8, Supplementary Figs 8D–F).

**Figure 5:**
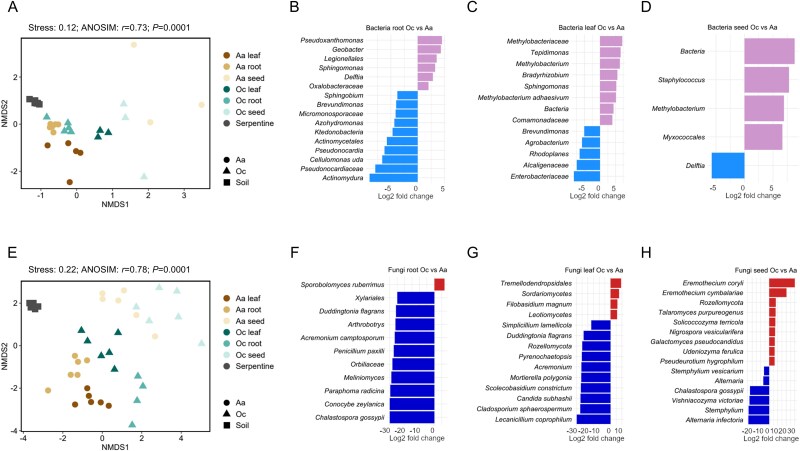
Assessing microbial community dissimilarity between *Arabidopsis arenosa* and *Odontarrhena chalcidica* through Dseq2 and NMDS analyses: nonmetric multidimensional scaling (NMDS) plot of Bray–Curtis dissimilarity indexes calculated on ASVs read counts from bacterial (A) and fungal (E) data (stress value for NMDS and ANOSIM test results were shown over the plot); DESeq analysis identifying up to 10 the most differentially abundant bacterial (B–D) and fungal (F-H) genera in root, leaf, and seed between plant species *A. arenosa* (Aa) and *Odontarrhena chalcidica* (Oc). ASVs to the left of the zero line are less abundant in Oc and ASVs to the right of the zero line are more abundant in Oc. All differentially abundant taxa are listed in the Supplementary Table 7 and 8.

NMDS ordination based on Bray–Curtis dissimilarity and ANOSIM confirmed significant differences in microbial composition between soil and plant endospheres (Fig. 5A, E, Supplementary Fig. 8). DESeq2 analysis identified several differentially abundant bacterial and fungal genera between plant species. *O. chalcidica* roots were enriched in *Pseudoxanthomonas* and *Geobacter*, whereas *A. arenosa* roots showed higher abundance of *Actinomadura* and *Pseudonocardiaceae*. Distinct genera also characterized leaves and seeds (Figs 5B–D).

Fungal communities also displayed species-specific patterns. *O. chalcidica* was enriched in *Sporobolomyces* in roots*, Filobasidium* in leaves and *Eremothecium* in seeds, whereas *A. arenosa* exhibited higher relative abundance of *Conocybe, Chalastospora* in roots*, Lecanicillium* in leaves*,* and *Stemphylium* in seeds (Figs 5F–H).

Functional predictions based on PICRUSt2 analysis indicated that the functional potential of root-associated bacterial communities in both *A. arenosa* and *O. chalcidica* closely resembled that of the serpentine soil, with shared enrichment in oxidative and redox metabolism pathways (Supplementary Fig. 10). Despite this overall similarity, the *A. arenosa* root microbiota showed a slightly higher predicted activity of energy metabolism–related functions (for more details refer to Supplementary results, Supplementary Table 10). Fungal guild analysis further revealed a predominance of saprotroph–symbiotroph fungi in roots and soil, whereas aboveground tissues exhibited more variable and host-specific functional profiles (for details refer to Supplementary Fig. 11, Supplementary results and Supplementary Table 11).

### Serpentine microorganisms drive species-specific shifts in metal transporter gene expression

**Figure 6:**
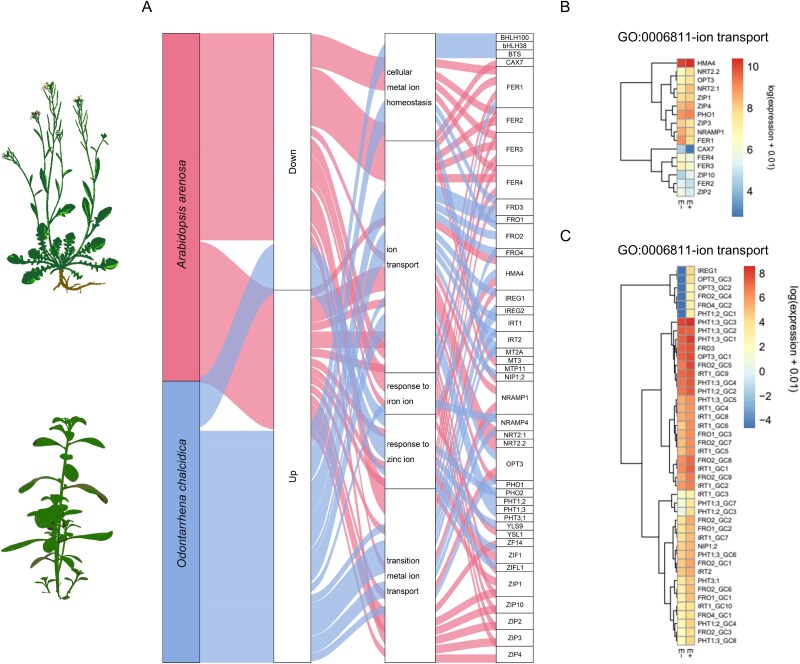
Species-specific metal transporter expression induced by serpentine microorganisms: (A) Sankey diagram illustrating the influence of serpentine soil extract inoculation (E+) on the expression of genes associated with five selected GO terms in *Odontarrhena chalcidica* and *Arabidopsis arenosa*, cultured *in vitro* for 7 days on medium supplemented with 500 μM Ni and 100 μM Mn. Gene expression changes were assessed relative to autoclaved soil extract inoculated plants. The experiment was conducted with five biological replicates per treatment; (B) heatmap showing the log of expression of genes annotated to the “ion transport” category (GO:0006811) in *A. arenosa* and *O. chalcidica* (C) inoculated with serpentine soil extract (E+) and sterile serpentine soil extract (E-). The remaining heatmaps for the selected categories are presented in Supplementary Fig. 12. All differentially expressed genes from these categories are listed in Supplementary Tables 12 and 13.

Metal uptake from the soil is governed by a coordinated network of metal transporters, whose expression is tightly regulated to meet the plant’s specific elemental requirements. We hypothesized that microorganisms may enhance metal uptake by modulating the expression patterns of plant metal transporters. To test this, we compared the root transcriptomes (RNA-seq) of *in vitro*–cultured *O. chalcidica* and *A. arenosa*, either inoculated with a serpentine-soil microbial extract or left uninoculated (inoculated with sterile extract). The analysis revealed distinct transcriptional responses to inoculation. Although only *O. chalcidica* showed a significant alteration in Ni accumulation, both species exhibited downregulation of all genes assigned to the “response to stress” GO category (GO:0006950), suggesting that soil microorganisms generally alleviate metal-induced stress in the host regardless of its inherent tolerance strategy (Fig. 6A, Supplementary Fig. 12A and E).

Focusing on Ni uptake in inoculated *O. chalcidica*, we observed upregulation of *IRT1* (AT4G19690) within the “nickel cation transport” category (GO:0015675). More broadly, genes involved in “ion transport” (GO:0006811), “cellular metal ion homeostasis” (GO:0006875), “response to iron ion” (GO:0010039) and “transition metal ion transport” (GO:0000041) were also induced. These included orthologs of *A. thaliana* iron-transporter genes—*IRT1, IRT2* (AT4G19680), *IREG1* (AT2G38460), *IREG2* (AT5G03570), *FRO1 (AT1G01590)*, FRO2 *(AT1G01580)*, *OPT3* (AT4G16370), *BTS* (AT3G18290), *bHLH38* (AT3G56970) and *NRAMP4* (AT5G67330) (Fig. 6A, C and Supplementary Figs 12A–D, Supplementary Table 12).

In contrast, *A. arenosa* roots showed downregulation of iron-transport genes but upregulation of genes related to zinc transport, including *ZIP4* (AT1G10970), *ZIP10* (AT1G31260), *ZIF1* (AT1G58340), *HMA4* (AT2G19110) and *ZIP1* (AT3G12750), all classified under “ion transport” (GO:0006811), “response to zinc ion” (GO:0010043) and “transition metal ion transport” (GO:0000041) (Fig. 6A and B, Supplementary Figs 12E–H, Supplementary Table 13). These divergent transcriptional signatures suggest that the two species employ different metal-transport pathways for adaptation to Ni excess.

## Discussion

Previous studies have demonstrated that microorganisms promote metal hyperaccumulation in plants by enhancing growth, supplying nutrients and modifying metal bioavailability through mechanisms such as siderophore production or pH alteration [11, 23, 40]. Contrary to these findings, our results show that microorganisms could enhance plant’s Ni uptake independently of growth promotion or increased Ni availability. The plant’s ability to accumulate nickel (Ni) was influenced by the specific microorganisms it interacted with. Microbiota from different origins, despite exhibiting comparable Ni-solubilizing capabilities, could either enhance or suppress Ni uptake in the same plant species. Our findings indicate that plant-associated microbiota regulate metal uptake and accumulation primarily by modulating the plant’s internal metal uptake mechanisms, rather than by altering Ni availability in the soil. In our model, the application of plant growth-promoting microorganisms activated plant growth without improving Ni accumulation (Fig. 2B and C). However, a serpentine-adapted microbial consortium enhanced both biomass production and Ni uptake (Fig. 2G, H, M, N) suggesting that growth promotion and Ni accumulation are independent processes. Furthermore, our *in vitro* studies confirmed that Ni uptake by plants is independent of metal availability (Fig. 3C, D, F, G). We therefore hypothesized that assembling an appropriate microbiota could optimize plant metal homeostasis, promoting hyperaccumulation and efficient Ni uptake. Indeed, *Odontarrhena chalcidica* co-cultured with an appropriate set of microorganisms accumulated significantly more Ni (Fig. 2H,N)—consistent with prior single-strain inoculation experiments showing that certain microorganisms can enhance Ni uptake in hyperaccumulators whereas inhibiting it in metal excluders [23, 41–43]. Conversely, we predicted that compatible microorganisms would suppress Ni uptake in the metal-excluding *A. arenosa*. Supporting this, our earlier work demonstrated that the endophytic yeast *Sporobolomyces ruberrimus*—known to confer metal tolerance in *Arabidopsis*—significantly reduced plant Ni accumulation [41]. However, *A. arenosa* co-cultured with the calamine SynCom (I1) took up more Ni than uninoculated controls (Fig. 2E and F), whereas the same SynCom had no effect on *O. chalcidica* (Fig. 2B and C). In contrast, *A. arenosa* grown in nonsterilized serpentine soil (with its native microbiota intact) exhibited a marked decrease in Ni uptake (Fig. 2E, F, K). We note, however, that our serpentine SynComs, being less diverse than the native soil community, may have lacked key taxa required to restrict Ni uptake in *A. arenosa*. Despite these variations in Ni accumulation, neither species exhibited altered growth or visible stress symptoms, suggesting that distinct microbial assemblages can modulate host tolerance strategies without compromising plant vigor (Fig. 2A, D, M). Further research is needed to unravel the mechanisms behind *A. arenosa’s* differential responses to SynComs. Nevertheless, our results clearly indicate that the composition of the surrounding microbial community decisively shapes plant metal-tolerance strategies.

Although microorganisms from the calamine SynCom possessed traits associated with enhancing Ni availability in soil (Fig. 1F and G, Supplementary Table 4) only the Ni-adapted consortium from serpentine soil led to increased Ni uptake in the *O. chalcidica*. Only microorganisms isolated from serpentine soils included strains capable of mobilizing Ni without producing siderophores or solubilizing phosphate, indicating a potential adaptation to elevated Ni levels in these environments (Fig. 1F and G). Despite increased Ni bioavailability in sterilized soil (Supplementary Table 6), serpentine-adapted microorganisms did not enhance Ni uptake by *A. arenosa* (Fig. 2K, L), indicating that the elevated Ni accumulation observed in *O. chalcidica* stems from its inherent absorption capacity rather than changes in soil Ni availability.

Heat, pressure, or microbial activity can induce alterations in metal availability, potentially resulting in variations in metal accumulation by plants [39, 44]. Autoclaving soil resulted in a substantial increase in bioavailable Mn, Ni, and Cu concentrations, significantly reducing the Ni:Mn concentration ratio (Supplementary Table 6). To account for this, we calculated the BF, which compares metal content in the plant with that in the soil. This approach allowed us to evaluate plant Ni uptake independently of metal availability in the soil. Plants inoculated with serpentine soil extract showed a higher BF for Ni than uninoculated controls, highlighting the important role of microbial interactions in hyperaccumulation (Fig. 3A and B). In contrast, microorganisms from calamine sites, unadapted to Ni excess, had no effect on the hyperaccumulator’s uptake (Fig. 2B and C) but did induce Ni accumulation in both roots and shoots of *A. arenosa* (Fig. 2E and F). These findings support the thesis that only species-specific microorganisms assist plant metal-tolerance strategy and thereby optimize metal uptake.

Metal uptake is regulated by both soil availability and by the plant’s internal metal homeostasis system, the optimal functioning of which is essential for vegetation in metal-rich environments. Microorganisms and their host plants co-adapt to optimize metabolism in specific environments [45]. *O. chalcidica* and *A. arenosa* recruited distinct microbial communities from serpentine soil (Fig. 4 and 5). *O. chalcidica* seemed to interact selectively with specific microorganisms, potentially those optimizing adaptation to the metal-rich environment. As shown previously [46], different plants release distinct organic compounds into the rhizosphere, which can shape microbial communities. This selective interaction may explain the divergence in plant responses to Ni uptake between *O. chalcidica* and *A. arenosa.* This also highlights the critical role of microorganisms in enhancing plant performance under metal stress.

The endosphere of *O. chalcidica* and *A. arenosa* was inhabited by a relatively high number of identical bacterial taxa. Additionally, a large fraction of these bacteria were shared with the soil (Fig. 4B). The plant mycobiome seemed to be less related to the soil, in the case of both plant species. Only a small fraction of fungi identified in the soil were found in the plant endosphere. The mycobiome of *A. arenosa* shared a significantly larger fraction of common fungi with the soil than the mycobiome of *O. chalcidica*; over half of the taxa identified in *A. arenosa* roots were present in the soil, and over 40% of fungal taxa identified in leaves were found in the soil, compared to only approx. 20% and 50% of the fungi found in *O. chalcidica* roots and leaves, respectively (Fig. 4E). This implies that the hyperaccumulator was more selective in its recruitment of fungi from the soil than *A. arenosa*; *O. chalcidica* assembled its mycobiota from sources other than the soil. Soil-borne microorganisms were responsible for activation of Ni uptake, indicating that sources other than the soil (seeds and air) do not play a role in this process. According to previously described results [47] the control of plant root colonization by fungi is a necessary condition for optimal plant function. Thus, we can imagine that the response of *O. chalcidica* to inoculation (and to culture in unsterilized soil) may have resulted from more efficient control of root colonization by the microorganism. This would imply that *O. chalcidica’s* ability to benefit from the soil microbiota was related to its ability to selectively interact with those that were potentially beneficial.

The most abundant microorganisms identified in both species were absent in the serpentine inoculum, suggesting that they were not directly responsible for enhanced Ni uptake. We cannot exclude that these microorganisms were present in the soil extract, which suggests some flexibility and redundancy in the function of different microbial taxa.

This selective recruitment of soil microorganisms by *O. chalcidica* is further reflected in the composition of its root microbiota compared to *A. arenosa*. DESeq2 analysis revealed several bacterial taxa enriched in *O. chalcidica*, including *Sphingomonas*, which are highly Ni-tolerant and, in some hyperaccumulators, can enhance Ni uptake via siderophore production or metal mobilization [11] (Fig. 5B). Other enriched bacteria, such as *Pseudoxanthomonas* [48, 49] and *Geobacter* [50], may influence Ni availability indirectly, although direct evidence for plant uptake is lacking. Among fungi, *Sporobolomyces ruberrimus* was enriched in *O. chalcidica*; related species are known to act as endophytes, protect plants from metal toxicity, regulate metal homeostasis, and immobilize metals such as iron, potentially limiting host exposure to toxic ions [41, 42, 51–54]. In contrast, taxa that were less abundant in *O. chalcidica* compared to *A. arenosa*, including bacterial *Brevundimonas* [55], *Pseudonocardiaceae* [56], *Actinomycetales* [44, 57] and fungal *Paraphoma radicina* [58, 59], are known from the literature to tolerate heavy metals, promote plant growth, or reduce metal toxicity, suggesting a potential role in metal tolerance in *A. arenosa*. Overall, these observations suggest that enriched taxa in *O. chalcidica* may facilitate Ni uptake, whereas less abundant taxa could contribute indirectly to metal tolerance in *A. arenosa*. Functional inference analyses further supported these taxonomic differences. PICRUSt2-based functional profiling of bacterial communities associated with both plant species and the serpentine soil revealed overlapping metabolic potentials between root and soil microbiota, with *A. arenosa* roots showing slightly higher predicted activity of general energy metabolism pathways, suggesting a broader metabolic capacity (Supplementary Fig. 10). Fungal functional guilds predicted using FUNGuild indicated that saprotroph-symbiotroph fungi dominated roots of both plant species and resembled the soil, whereas leaves and seeds showed more variable guild composition. *A. arenosa* roots harbored relatively higher abundances of pathotroph and symbiotroph fungi compared *with O. chalcidica*, consistent with a greater fungal functional potential (Supplementary Fig. 11). More studies are needed to confirm causal effects and clarify the specific contributions of individual microbial taxa.

Inoculation of *O. chalcidica* with serpentine-soil extract triggered a robust induction of iron-transporter genes (Fig. 6A, C), reinforcing earlier observations that this species shares its Fe-transport machinery for Ni acquisition. It was previously shown [60] that *O. chalcidica* can accumulate exceptionally high Zn levels in its roots when grown in Zn-rich media, and that Zn suppresses Ni uptake whereas Ni has minimal impact on Zn accumulation [61]. Together, these findings support a model in which low-affinity Fe and Zn transporters serve as the primary entry routes for Ni in *O. chalcidica*. In our experiment, microbial inoculation selectively amplified Fe-transporter expression, rather than Zn-specific carriers, suggesting that serpentine microbiota enhance Ni uptake by enhancement of the plant’s iron-transport pathway. By contrast, *A. arenosa* roots responded to the same inoculum with upregulation of zinc-transporter genes and the nitrate transporters *NRT2.1, NRT2.2*, recently linked to serpentine adaptation [22, 62] (Fig. 6A and B). This transcriptional profile reflects the rapid edaphic adaptation documented up to date [21], wherein *A. arenosa* populations fine-tune micronutrient transport to thrive under high Ni concentrations. Altogether, our data suggest that serpentine-derived microorganisms reinforce each species’ inherent metal-handling strategy*: O. chalcidica* enhances Ni uptake through its iron-transport system, whereas *A. arenosa* prioritizes cation partitioning strategies that mitigate Ni stress.

In conclusion, our findings clearly show that the assembly of an appropriate microbiota allows plants to optimize their adaptation to the environment. This microbial community appeared to enhance plant metal uptake by improving plant metal homeostasis rather than simply increasing Ni bioavailability in the soil, highlighting the importance of plant-microbe interactions in metal-rich environments.

## Supplementary Material

Supplementary_Table_1_wraf265

Supplementary_Table_2_wraf265

Supplementary_Table_3_wraf265

Supplementary_Table_4_wraf265

Supplementary_Table_5_wraf265

Supplementary_Table_6_wraf265

Supplementary_Table_7_wraf265

Supplementary_Table_8_wraf265

Supplementary_Table_9_wraf265

Supplementary_Table_10_wraf265

Supplementary_Table_11_wraf265

Supplementary_Table_12_wraf265

Supplementary_Table_13_wraf265

Supplementary_Methods_wraf265

Supplementary_Results_wraf265

## Data Availability

The data generated in the high-throughput sequencing are available in the Sequence Read Archive (SRA, National Center for Biotechnology Information) repository under BioProject ID PRJNA1177665. The RNAseq data are available in the SRA repository under BioProject ID PRJNA1333822.
