# Supplementary material for: Regulation of plant Ni uptake by soil-borne microorganisms occurs independently of their Ni-solubilizing capabilities
Source: ISME J. 2025 Dec 1;19(1):wraf265. doi: 10.1093/ismejo/wraf265 (PMC12746290; doi:10.1093/ismejo/wraf265)
Supplement: Supplementary_Methods_wraf265 [file supplementary_methods_wraf265.pdf]

Supplementary Information for

Regulation of plant Ni uptake by soil-borne microorganisms occurs independently of their Ni-solubilizing capabilities

Agnieszka Domka [1, 2*], Maciej Gustab [2,3], Roman J. Jędrzejczyk [2], Rafał Ważny [2], Alice Tognacchini [4], Markus Puschenreiter [4], Paweł Łabaj[2], Agata Muszyńska[2], Weronika Kosowicz [2,3], Kinga Jarosz[5], Piotr Rozpądek[2*]

[1] Polish Academy of Sciences, W. Szafer Institute of Botany, Lubicz 46, 31-512 Kraków, Poland
[2] Malopolska Centre of Biotechnology, Jagiellonian University in Kraków, Gronostajowa 7a, 30-387 Kraków, Poland
[3] Jagiellonian University in Kraków, Doctoral School of Exact and Natural Sciences, Łojasiewicza 11, 30-348 Kraków, Poland
[4] University of Natural Resources and Life Sciences, Vienna, Department of Forest and Soil Sciences, Institute of Soil Research, Konrad-Lorenz Straße 24, 3430 Tulln, Austria
[5] Institute of Geological Sciences, Faculty of Geography and Geology, Jagiellonian University, ul. Gronostajowa 3a, 30-387 Kraków, Poland
** corresponding author*

Experimental design

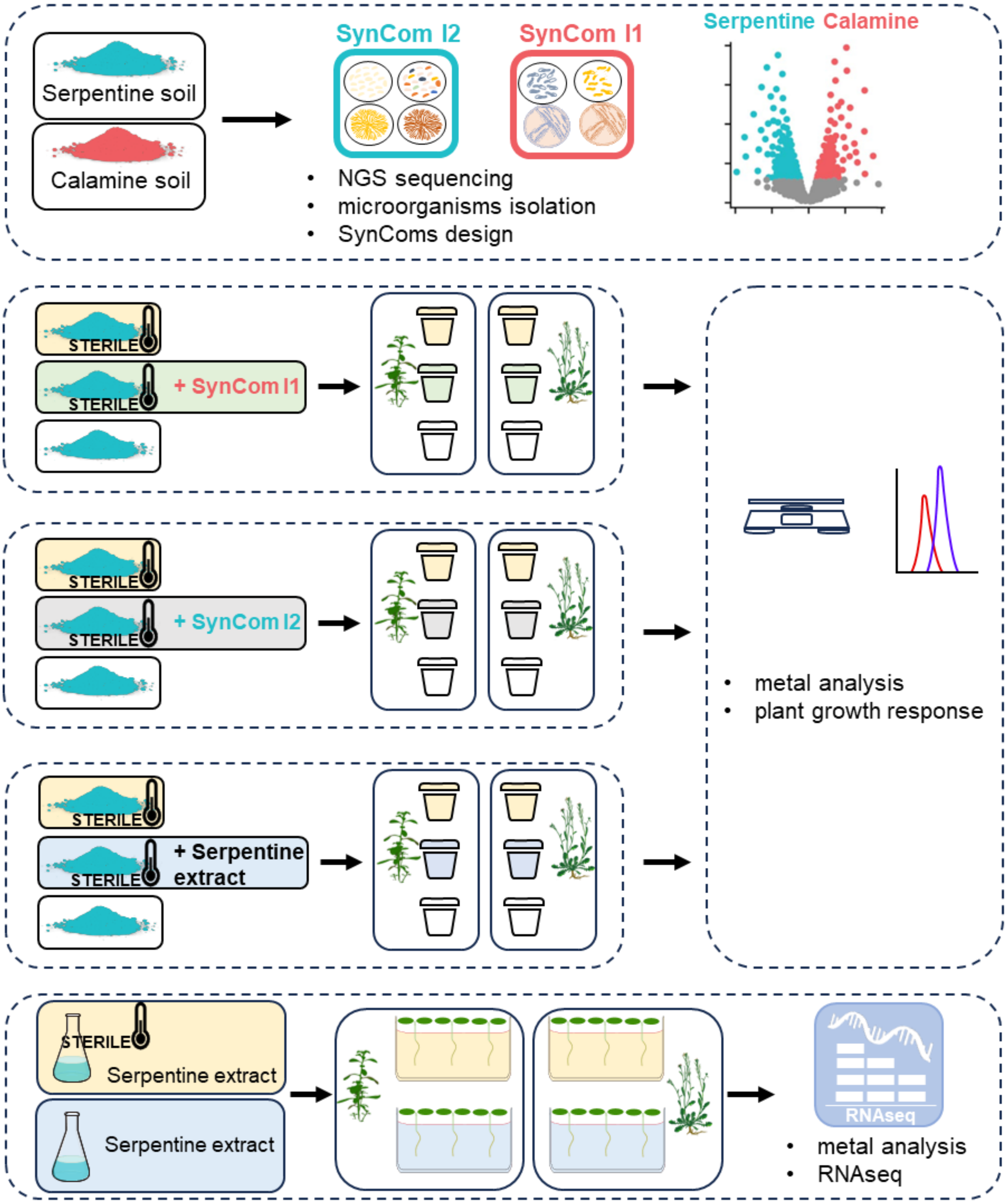

Supplementary Materials and Methods

Chemical analysis of the soil

The soil was sieved through a standardized sieve made of metal-free material with a mesh size of 2 mm. It was then divided into subsamples for analysis.

Water Content: The dry mass was measured at 105°C in the oven (POL-EKO, PL).

Soil pH: The pH of a soil suspension in water and in $CaCl_2$ (soil ratio of 1:5 v/v) was measured using a calibrated pH meter (ELMETRON CP-105, Poland).

Water Content Capacity [1]: Approximately 50 mL soil samples were saturated with deionized water and left for 72 hours to ensure full saturation. The samples were then placed on sand with a coarse grain size for 20 minutes. After that, they were dried in an oven at 105°C for 12 hours. The water capacity was determined by measuring the change in the net mass of the soil.

Organic Matter Content: Dried soil samples at 105°C were placed in ceramic crucibles and heated in an oven at 550°C for 6 hours. After cooling in a desiccator, the mass was determined. The difference in mass represents the organic matter content.

Kjeldahl Nitrogen Concentration: Soil samples were dried at 40 °C in an oven. Test samples (1 g) were prepared with the accuracy of 0.0001 g. A mixture of 98% sulfuric acid and 4 mL of salicylic acid was added, and the samples were predigested for 12 hours. Sodium thiosulfate (0.5 g) was then added. After cooling, 1.1 g of a catalytic mixture (copper(II) sulfate pentahydrate, potassium sulfate, and titanium dioxide in proportions of (w/w) 6 : 200 :6 g) was added and heated at 350°C for 2 hours. After cooling, 10 mL of deionized water and 5 mL of boric acid were added, followed by steam distillation. The distillate was titrated with 0.01 M hydrochloric acid using an indicator - mixture of bromocresol green and methyl red.

Total Phosphorus: Soil samples were digested with 98% sulfuric acid, and phosphorus concentration was determined using UV–Vis spectroscopy using an external calibration method.

Labile Phosphorus (according to previously established method [2] with modifications): Five grams of dried soil (with an accuracy of 0.001 g) were placed in an Erlenmeyer flask with 1 g of activated carbon. 100 mL of 0.5 M $NaHCO_3$ (pH = 8.5) extraction solution was added, and the mixture was shaken for 30 minutes at 100 rpm. The suspension was filtered (phosphorous – free filter, and phosphorus concentration in the extracts was measured using UV–Vis spectroscopy.

Metal Concentration: Soil samples were immersed in 10 mL of a nitric acid and hydrochloric acid mixture (1:3 v/v) and left to predigest overnight. The suspension was then boiled for 2 hours, cooled, and filtered through a metal-free, phosphorus-free paper filter. The concentrations of individual elements were determined using flame atomic absorption spectrometry (FAAS), Graphite Furnace Atomic Absorption Spectrometry (GFAAS) using an external standard calibration curve to ensure accurate quantification of the target elements.

Plant growth promoting properties of bacteria and fungi

Phosphate solubilization: Bacterial and fungal phosphate-solubilizing potential was assessed by culturing them on Pikovskaya [3] medium for 7 days at 30°C in darkness (N=3). Bacteria were grown on solid Pikovskaya medium, while fungi were cultured in liquid medium. Tri-calcium phosphate served as the source of insoluble phosphate. Phosphate solubilization was indicated by a clear zone surrounding bacterial colonies on solid medium and by the clearing of the liquid medium for fungal cultures.

Siderophore production: To evaluate siderophore production, a modified version of the chromeazurol S (CAS) blue agar method [4] was used. Four solutions (1-4) were prepared and combined in the order: 2, 3, 4, 1, then poured into plates. Solution 1: 100 mL dd H_2O, 2.7 g $FeCl_3 \cdot 6H_2O$, 180 µL HCl (0.56 mM), 60.5 g chromeazurol S (CAS) and 72.8 mg HDTMA bromide, autoclaved; Solution 2: 800 mL dd H_2O_2, 0.3 g KH_2PO_4, 0.5 g NaCl, 1 g NH_4Cl, 30.24 g PIPES (to dissolve PIPES pH was adjusted to 6.8) and 15 g agar, autoclaved; Solution 3: 70 mL dd H_2O, 2 g glucose, 2 g mannitol, 0.493 g $MgSO_4 \cdot 7H_2O$, 11 mg $CaCl_2 \cdot 2H_2O$, 1.17 mg $MnSO_4 \cdot H_2O$, 1.4 mg H_3BO_4, 0.04 mg $CuSO_4 \cdot 5H_2O$, 1.2 mg $ZnSO_4 \cdot 7H_2O$, 1 mg $Na_2MoO_4 \cdot 2H_2O$, autoclaved; Solution 4: 3 g hydrate of casein was dissolved in 30 mL dd H_2O_2 and filter sterilized. Bacterial and fungal cultures, along with *Pseudomonas protegens* as a positive control, were grown on CAS blue agar for 14 days, with daily monitoring. Microorganisms capable of producing siderophores removed iron from the dye complex, resulting in a color change in the medium from blue to orange.

Ni mobilization: The ability of bacterial and fungal isolates to mobilize Ni was evaluated following the published method [5], with minor modifications. Bacterial strains were cultured in 25 mL of LB medium at 30°C with shaking (180 rpm) until reaching late logarithmic phase. Cells were harvested by centrifugation (12 000 × g, 20°C, 10 min), washed twice with sterile 10 mM $MgSO_4$, and resuspended in the same buffer to an OD_{600} of 0.5. One milliliter of each suspension was inoculated into 2 g of autoclaved serpentine soil in 15 mL glass tubes. Control tubes contained 2 g of sterile soil supplemented with 2 mL of sterile 10 mM $MgSO_4$.
For fungal assays, a mycelial plug (~5 mm diameter) from a 5-day-old culture on PDA was transferred into 2 g of sterile serpentine soil suspended in 2 mL of sterile 10 mM $MgSO_4$. All samples, including controls, were prepared in triplicate. Tubes were incubated at room temperature with shaking (180 rpm) for 7 days. After incubation, 8 mL of deionized water was added to each tube, followed by centrifugation (4 500 × g, 20°C, 5 min). Supernatants were filtered, and Ni concentrations were determined as described in the Supplementary methods (Chemical analysis of soil).

Isolation and identification of microorganisms from serpentine soil

To isolate microorganisms from serpentine soil, five freshly sieved soil samples (approximately 1g each) were suspended in 10 mL of sterile 0.9% NaCl solution. These 10^{-1} diluted samples were serially diluted to concentrations of 10^{-2}, 10^{-3} and 10^{-4}. For fungal isolation, 100 µL of the 10^{-1} dilution was spread onto PDA plates supplemented with antibiotics (200 mg · L^{-1} of ampicillin, 100 mg · L^{-1} of tetracycline and 200 mg · L^{-1} of streptomycin) to inhibit bacterial growth, in triplicate. To isolate bacteria, 100 µL of the 10^{-4} dilution was spread onto plates containing 1/10 869 medium [5] and nutrient agar (NA), also in triplicate. Colony formation was monitored after 1 day for bacteria and after 7 days for fungi. Pure cultures were obtained by subculturing the isolated bacteria and fungi on their respective media and these were subsequently used for identification.

Fungi and bacteria were identified through their morphological traits and by sequencing specific genetic regions, including the internal transcribed spacer 1 (ITS1), the 5.8S rRNA gene, the

internal transcribed spacer 2 (ITS2), and the large subunit of rRNA (LSU). DNA extraction was performed using the cetyltrimethylammonium bromide (CTAB) method, following the published protocol [6], with modifications previously described [7].

For fungal identification, the ITS-LSU rRNA was amplified using ITS1F and LR5 primers [8, 9]. For bacterial identification the 16 S rRNA region was amplified with the primers 27F and 1492R [10]. Polymerase chain reaction (PCR) was conducted in 25 µL reaction mixtures, including 5–10 ng of DNA, 9.5 µL nuclease-free water, 12.5 µL DreamTaq HS Green PCR Master Mix, and 1 µL of each primer at 10 pmol. PCR products were visualized on a 1.5% agarose gel stained with GelRed. After purification using the EPPiC Fast kit, the products were sequenced by Macrogen Europe Laboratory (NL). Sequences were edited in Geneious Prime (www.geneious.com) and compared to those in the NCBI (National Centre for Biotechnology Information) database using BLASTn (www.ncbi.nlm.nih.gov). Fungal and bacterial species were identified with ≥98% sequence similarity, and sequences were submitted to NCBI under accession numbers listed in Supplementary table 1.

Plant and soil processing for high-throughput sequencing

Plant tissues (shoots and roots) were harvested, fractionated, and surface-sterilized by three centrifuge washes in sterile deionized water (1 min, 15,000 g), followed by treatment with 0.5% sodium hypochlorite containing Tween (3 min), rinsing in deionized water (2 min), 75% ethanol (30 s), and sterile deionized water (2, 5, and 1 min).

Seeds (50 mg each) were surface-sterilized by sequential immersion in deionized water (1 min), 70% ethanol (30 s), 1% sodium hypochlorite with Tween (1 min), 70% ethanol (30 s), and five rinses in sterile water (5 × 30 s). Seeds were homogenized in 200 µL of 10 mM $MgSO_4$ using a micropestle and used for DNA extraction.

Total DNA was extracted using a modified CTAB protocol based on well-established protocol [11]. Briefly, 50 mg of plant tissue was homogenized and incubated with 700 µL CTAB buffer and 20 µL 1 M PVP at 65°C for 15 min (300 rpm). Proteinase K (20 µL) was added, followed by further incubation (30 min at 65°C, then 10 min at 95°C). Samples were centrifuged (30 min, 15,000 g), and the supernatant was extracted twice with chloroform. DNA was precipitated with isopropanol and incubated overnight at −80°C, centrifuged (45 min, 15,000 g, 4°C), washed twice with 70% ethanol, air-dried (10 min, 55°C), and resuspended in 50 µL TE buffer. DNA quantity and purity were assessed spectrophotometrically.

Soil DNA was extracted from 5 g of soil using a modified CTAB-based protocol [6], followed by purification with the MagMAX™ Microbiome Ultra Nucleic Acid Isolation Kit (Thermo Scientific).

SEM-EDX analysis

Fresh leaves of *O. chalcidica* from in vitro cultures were mounted on SEM sample holders, freeze-dried at –80°C, and subsequently examined using SEM/EDS. Scanning electron microscopy (SEM) was conducted using a Hitachi SU8600 ultra-high-resolution field emission scanning electron microscope (FE-SEM) at the Scanning Electron Microscopy and Microanalysis Laboratory, Institute of Geological Sciences, Jagiellonian University. Both secondary electron (SE) and back-scattered electron (BSE) signals were used for image acquisition. Energy-dispersive X-ray spectroscopy (EDS) analyses for chemical composition were carried out with a Bruker XFlash 7 detector and QUANTAX FlatQUAD system for elemental mapping. EDS measurements were performed at an accelerating voltage of 20 keV, a beam current of 10 µA, and a working distance of 13–15 mm. Elemental maps were collected over a 30-minute integration period.

Orthology and Gene-Level Mapping

To facilitate robust cross-species comparisons, TAIR10 orthologs were assigned based on KEGG annotations extracted from the Trinotate output. In *O. chalcidica*, de novo assembly coupled with redundancy filtering often produces multiple transcript variants that map to a single TAIR10 locus. This phenomenon can arise from several sources:

- Transcript fragmentation: Long or structurally complex transcripts may be assembled as discrete contigs rather than a single continuous sequence.

- Paralogous expansions and tandem gene clusters: Unlike the single-copy genes found in *A. thaliana, O. chalcidica* may harbor multiple, closely related paralogs or cluster duplications that correspond to one reference gene.

- Partial, non-overlapping homology: Independent assembly fragments may each align to distinct regions of the same reference gene.

Rather than collapsing these variants into a single representative, we preserved each transcript independently throughout differential expression and GO term enrichment analyses, thereby retaining subtle yet potentially significant expression differences. To ensure clear identification in downstream visualizations—such as heatmaps and network diagrams—each *O. chalcidica* transcript mapping to the same TAIR10 ortholog has been renamed using the convention <TAIR_ID>_GCn, where "GC" denotes "gene cluster" and n is a sequential index assigned in order of discovery.

Interpretation of GO Term Enrichment

GO enrichment results were interpreted with appropriate caution, acknowledging the limitations inherent to *de novo* transcriptome annotation. Although some functional assignments may be incomplete or ambiguous, the enrichment analysis compares DEGs against a background of all annotated genes from the same pipeline. Therefore, even if gene-level annotations are imperfect, relative enrichment statistics remain valid, since both the foreground (DEGs) and the background (gene universe) derive from the same annotation framework.

PICRUSt2 functional analysis

Predicted functional profiles of root- and soil-associated bacterial communities were generated using PICRUSt2 [12]. The unstratified KO table was imported into R and combined with sample metadata. Data were normalized to relative abundances, and singletons were checked to ensure data quality. Alpha diversity (Shannon and Simpson indices) and beta diversity (Bray–Curtis dissimilarity) were calculated using the phyloseq and vegan packages, with differences between groups assessed via Kruskal–Wallis tests and PERMANOVA, respectively.

Differentially abundant KOs among experimental groups were identified using Kruskal–Wallis tests with Benjamini–Hochberg correction. The top 20 most significantly different KOs were extracted, and their relative abundances were averaged by group to generate a Z-score-scaled matrix. Finally, heatmaps of top KOs across groups were produced using the pheatmap package, with hierarchical clustering applied to both rows (functions) and columns (groups).

FUNGuild functional analysis

Fungal functional profiles were predicted using FUNGuild based on the ASVs table obtained from ITS amplicon sequencing. The ASVs table was combined with sample metadata (SampleID and experimental group). Prior to analysis, ASVs counts were transformed to relative abundances within each sample (% of total reads per sample). Multi-mode assignments and inconsistent category labels (e.g., variant dashes, duplicate modes) were standardized and merged for consistency. Guilds detected in fewer than three samples were excluded, and optional filtering based on confidence ranking was available. For each functional category, the mean relative abundance per experimental group was calculated, and statistical differences between groups were assessed using Kruskal–Wallis tests followed by Dunn's post hoc tests with Bonferroni correction. Visualizations included stacked bar plots of mean relative abundances per group highlighting variation and significance of top trophic modes.

References

1. Wilke B M. Determination of Chemical and Physical Soil Properties. In: Monitoring and Assessing Soil Bioremediation. Soil Biology, vol 5. Springer, Berlin, Heidelberg. 2005. https://doi.org/10.1007/3-540-28904-6_2.
2. Olsen SR. Estimation of available phosphorus in soils by extraction with sodium bicarbonate. US Department of Agriculture, 1954.
3. Pikovskaya R. Mobilization of phosphorus in soil in connection with vital activity of some microbial species. *Microbiology* 1948;**17**: 362-370.
4. Schwyn B, Neilands JB. Universal chemical assay for the detection and determination of siderophores. *Anal Biochem* 1987;**160**:47–56. https://doi.org/10.1016/0003-2697(87)90612-9
5. Mergeay M et al. *Alcaligenes eutrophus* CH34 is a facultative chemolithotroph with plasmid-bound resistance to heavy metals. *J Bacteriol* 1985;**162**:328–334. https://doi.org/10.1128/jb.162.1.328-334.1985
6. Azmat MA et al. Extraction of DNA suitable for PCR applications from mature leaves of Mangifera indica L. *Journal of Zhejiang University Science B* 2012;**13**:239–243. https://doi.org/10.1631/jzus.B1100194
7. Domka A. *Mucor* sp. — An endophyte of *Brassicaceae* capable of surviving in toxic metal-rich sites. *J Basic Microbiol* 2019;**59**(1):24–37. https://doi.org/10.1002/jobm.201800406
8. Gardes M, Bruns TD. ITS primers with enhanced specificity for basidiomycetes -application to the identification of mycorrhizae and rusts. *Mol Ecol* 1993;**2**(2):113-8. https://doi.org/10.1111/j.1365-294x.1993.tb00005.x.
9. Vilgalys R, Hester M. Rapid genetic identification and mapping of enzymatically amplified ribosomal DNA from several *Cryptococcus* species. *J Bacteriol* 1990;**172**(8):4238-4246. doi:10.1128/jb.172.8.4238-4246.1990
10. Turner S et al. Investigating deep phylogenetic relationships among *Cyanobacteria* and plastids by small subunit rRNA sequence analysis. *J Eukaryot Microbiol.* 1999;**46**(4):327-338. doi:10.1111/j.1550-7408.1999.tb04612.x
11. Azmat MA et al. Extraction of DNA suitable for PCR applications from mature leaves of *Mangifera indica* L. *J Zhejiang Univ Sci B* 2012;**13**(4):239-243. doi:10.1631/jzus.B1100194
12. Douglas GM, Maffei VJ, Zaneveld JR et al. PICRUSt2 for prediction of metagenome functions. *Nat Biotechnol* 2020;**38**(6):685–688. https://doi.org/10.1038/s41587-020-0548-6
