# Supplementary material for: Regulation of plant Ni uptake by soil-borne microorganisms occurs independently of their Ni-solubilizing capabilities
Source: ISME J. 2025 Dec 1;19(1):wraf265. doi: 10.1093/ismejo/wraf265 (PMC12746290; doi:10.1093/ismejo/wraf265)
Supplement: Supplementary_Results_wraf265 [file supplementary_results_wraf265.pdf]

Supplementary Information for

Regulation of plant Ni uptake by soil-borne microorganisms occurs independently of their Ni-solubilizing capabilities

Agnieszka Domka [1, 2*], Maciej Gustab [2,3], Roman J. Jędrzejczyk [2], Rafał Ważny [2], Alice Tognacchini [4], Markus Puschenreiter [4], Paweł Łabaj[2], Agata Muszyńska[2], Weronika Kosowicz [2,3], Kinga Jarosz[5], Piotr Rozpądek [2*]

[1] Polish Academy of Sciences, W. Szafer Institute of Botany, Lubicz 46, 31-512 Kraków, Poland
[2] Malopolska Centre of Biotechnology, Jagiellonian University in Kraków, Gronostajowa 7a, 30-387 Kraków, Poland
[3] Jagiellonian University in Kraków, Doctoral School of Exact and Natural Sciences, Łojasiewicza 11, 30-348 Kraków, Poland
[4] University of Natural Resources and Life Sciences, Vienna, Department of Forest and Soil Sciences, Institute of Soil Research, Konrad-Lorenz Straße 24, 3430 Tulln, Austria
[5] Institute of Geological Sciences, Faculty of Geography and Geology, Jagiellonian University, ul. Gronostajowa 3a, 30-387 Kraków, Poland
** corresponding author*

Supplementary Results

DESeq2 analysis for *O. chalcidica* and *A. arenosa*

Figures 5 c-h showed up to ten the most differentially abundant bacterial and fungal genera in roots, leaves and seeds of *A. arenosa* and *O. chalcidica* analysed by DESeq. All differentially abundant taxa are listed in the Supplementary Table 6 and 7. Samples from *O. chalcidica* had significantly higher abundances of bacteria *Pseudoxanthomonas, Paenibacillus, Geobacter, Methylobacteriaceae, Legionellales, Sphingomonas, Caulobacter, Delftia, Propionibacterium* and *Methylobacterium* in the roots, *Tepidimonas, Methylobacterium, Methylobactertiaceae, Bradyrhyzobium, Sphingomonas, Comamonadaceae* and unidentified bacteria in the leaves, *Staphyllococcus, Betaproteobacteria, Corynebacterium*, *Myxococcales, Methylobacterium, Methylobactertiaceae* and unidentified bacteria in the seeds than samples from *A. arenosa*, where significantly higher abundances were shown by *Micromonosporaceae, Cryptosporangium, Sphingobium, Azohydromonas, Ktedonobacteria, Pseudonoccardia, Actinomycetales, Cellulomonas, Pseudonocardiaceae, Actinomadura* in the roots, *Stenotrophomonas, Nocardioides, Microbacteriaceae, Brevundimonas, Rhodoplanes, Alcaligeneceae* and *Enterobacteriaceae* in the leaves and *Delftia* in the seeds (Fig. 5 c-e).

Among the top abundant fungal endophytes in *O. chalcidica* there were *Sporobolomyces* and *Peniophora* in roots, *Ceratobiasidiaceae, Mortiellaceae, Filobasidium, Tremellodendropsidales, Sordariomycetes,* and *Meliniomyces* in leaves and *Eremothecium, Solicozyma* and *Rozellomycota* in the seeds. In samples of *A. arenosa* the highest significant abundances were shown for *Nectriaceae, Malassezia, Emericellopsis, Scolecobasidium, Periconia, Sarocladium, Subulicystydium, Gymnostellatospora, Arthrobotrys* and *Dominikia* in roots, *Nectriaceae, Neodevriesia, Didymellaceae, Conocybe, Niesslia, Nectriella, Penicillium, Stagonosporopsis, Fusarium* and *Scolecobasidium* in leaves and *Sporobolomyces, Vishniacozyma, Mortierellaceae, Nectriaceae, Filobasidium, Alternaria, Chalastospora, Stemphylium* in the seeds (Fig. 5 f-h).

Supplementary Figures

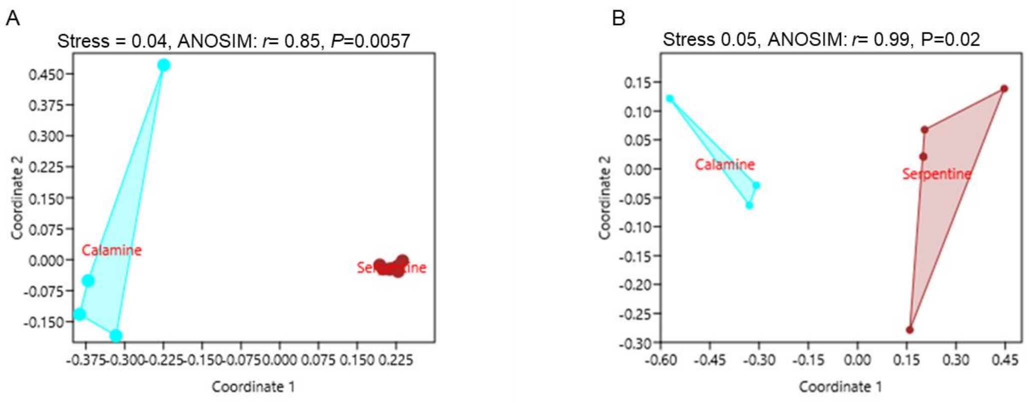

Supplementary fig. 1 Soil microbial diversity. Non-Metric Multidimensional Scaling (NMDS) plot of Bray-Curtis distances calculated on ASVs read counts from fungal (A) and bacterial (B) data (ANOSIM test results were shown over the plot)

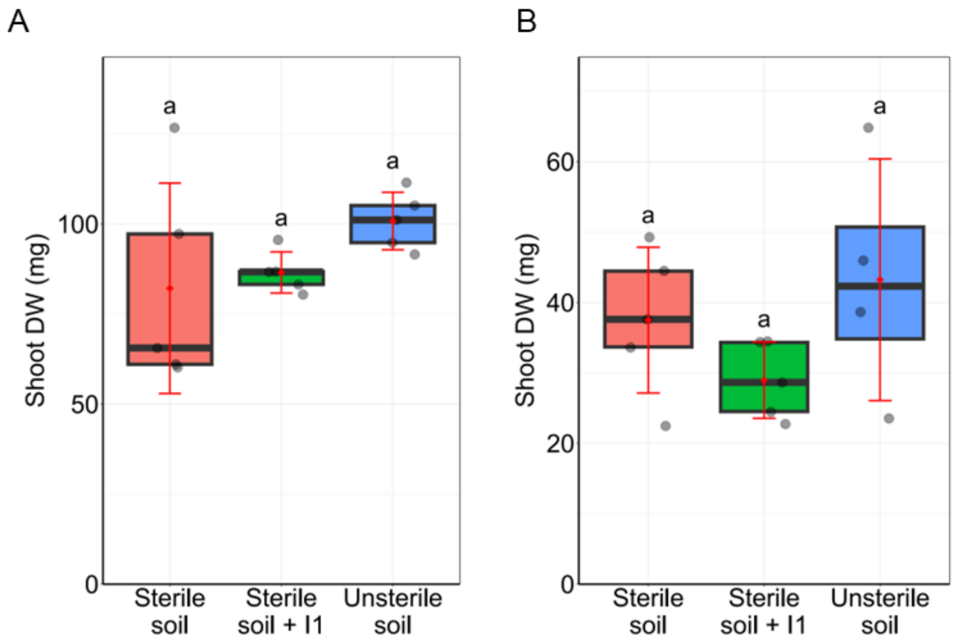

Supplementary fig. 2 Microbial influence on shoot biomass of *Odontarrhena calcidica* and *Arabidopsis arenosa.* Dry weight of *O. chalcidica* (A) and *A. arenosa* (B) shoots grown in sterilized serpentine soil (Sterile soil), inoculated with inoculum with microorganisms isolated from calamine mine dump (Sterile soil + **I1**) and grown in unsterile serpentine soil (Unsterile soil) (N=5, one plant per pot). Boxplots represent the median and interquartile range with standard deviation indicated in red. Letters above bars denote significant differences between groups, as assessed by one-way ANOVA with Tukey's post hoc test for all multiple comparisons ($P \leq 0.05$).

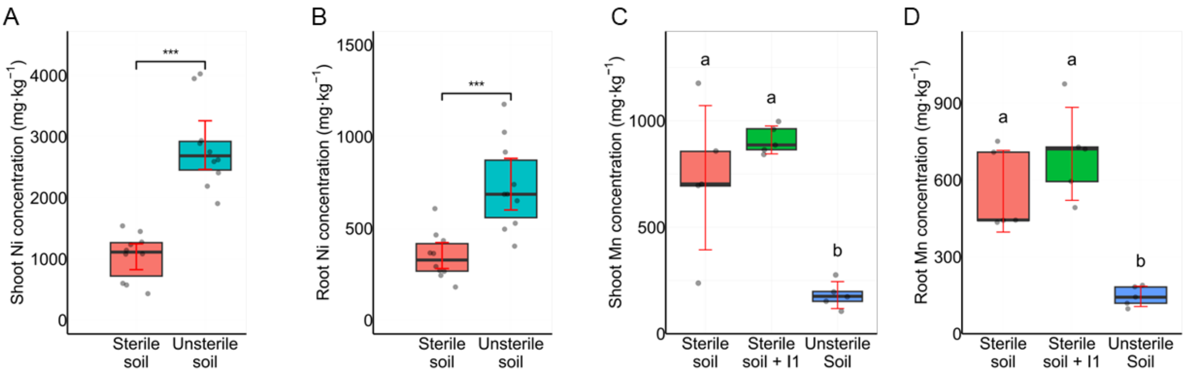

E

	BF_{Mn}	
	Root	Shoot
Unsterile soil	0.014 ± 0.004 b	0.031 ± 0.009 b
Sterile soil	0.003 ± 0.001 a	0.01± 0.003 a
Sterile soil + I1	0.006 ± 0.001 a	0.014 ± 0.003 a

Supplementary fig. 3 Microbial influence on Ni and Mn uptake in *Odontarrhena chalcidica*, (A, B) Ni concentration in shoots (A) and (B) roots of *O. chalcidica* grown in sterilized serpentine soil (Sterile soil) and in unsterile serpentine soil (Unsterile soil) (N=10) measured by FAAS. (C, D) Mn concentration in shoots (C) and roots (D) of *O. chalcidica* grown in Sterile soil, inoculated with inoculum with microorganisms isolated from calamine mine dump (Sterile soil + I1) and grown in Unsterile soil (N=5). (E) Mn bioconcentration factor (BF) calculated for roots and shoots of *O. chalcidica* plants grown in Sterile soil, Sterile soil + I1, and Unsterile soil. Boxplots represent the median and interquartile range with standard deviation indicated in red. Stars indicate statistically significant differences (t-test, * - $P \leq 0.05$, ** - $P \leq 0.01$, *** - $P \leq 0.001$) for pairwise comparisons and letters above bars denote significant differences between groups, as assessed by one-way ANOVA with Tukey's post hoc test for all multiple comparisons ($P \leq 0.05$). In all experiments, one plant was grown per pot.

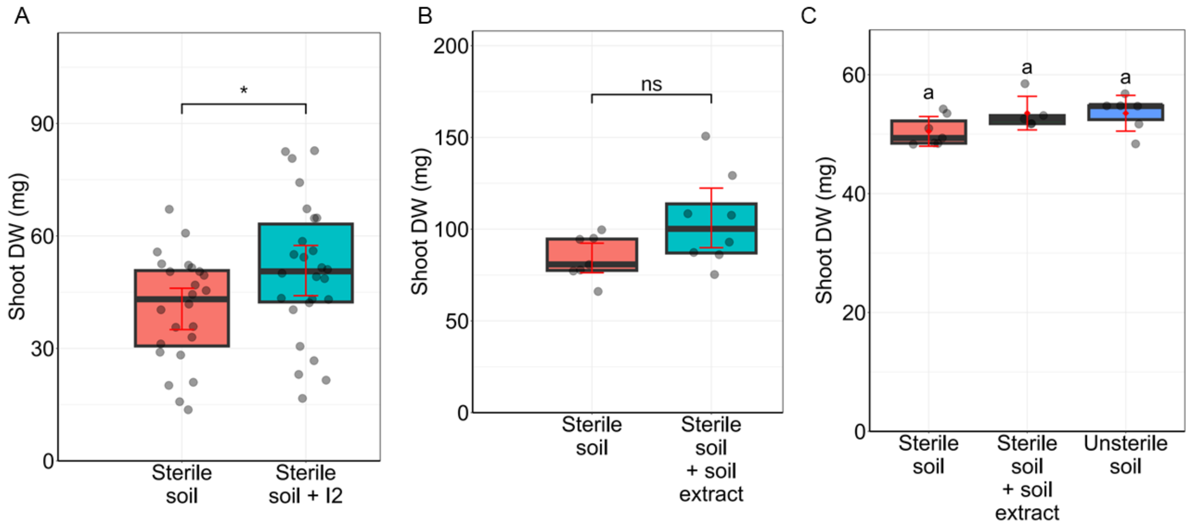

Supplementary fig. 4 Serpentine microorganisms influence on *Odontarrhena chalcidica* and *Arabidopsis arenosa* biomass. (A) dry weight of *O. chalcidica* shoots grown in sterilized serpentine soil (Sterile soil), inoculated with inoculum with microorganisms isolated from serpentine soil (Sterile soil + **I2**) (N=23-25). (B, C) dry weight of *O. chalcidica* (B) and *A. arenosa* (C) shoots grown in Sterile soil, sterilized serpentine inoculated with serpentine soil extract and in Unsterile soil (N=6-8). Boxplots represent the median and interquartile range with standard deviation indicated in red. Stars indicate statistically significant differences (t-test, * - $P \leq 0.05$, ** - $P \leq 0.01$, *** - $P \leq 0.001$) for pairwise comparisons and letters above bars denote significant differences between groups, as assessed by one-way ANOVA with Tukey's post hoc test for all multiple comparisons ($P \leq 0.05$). In all experiments, one plant was grown per pot.

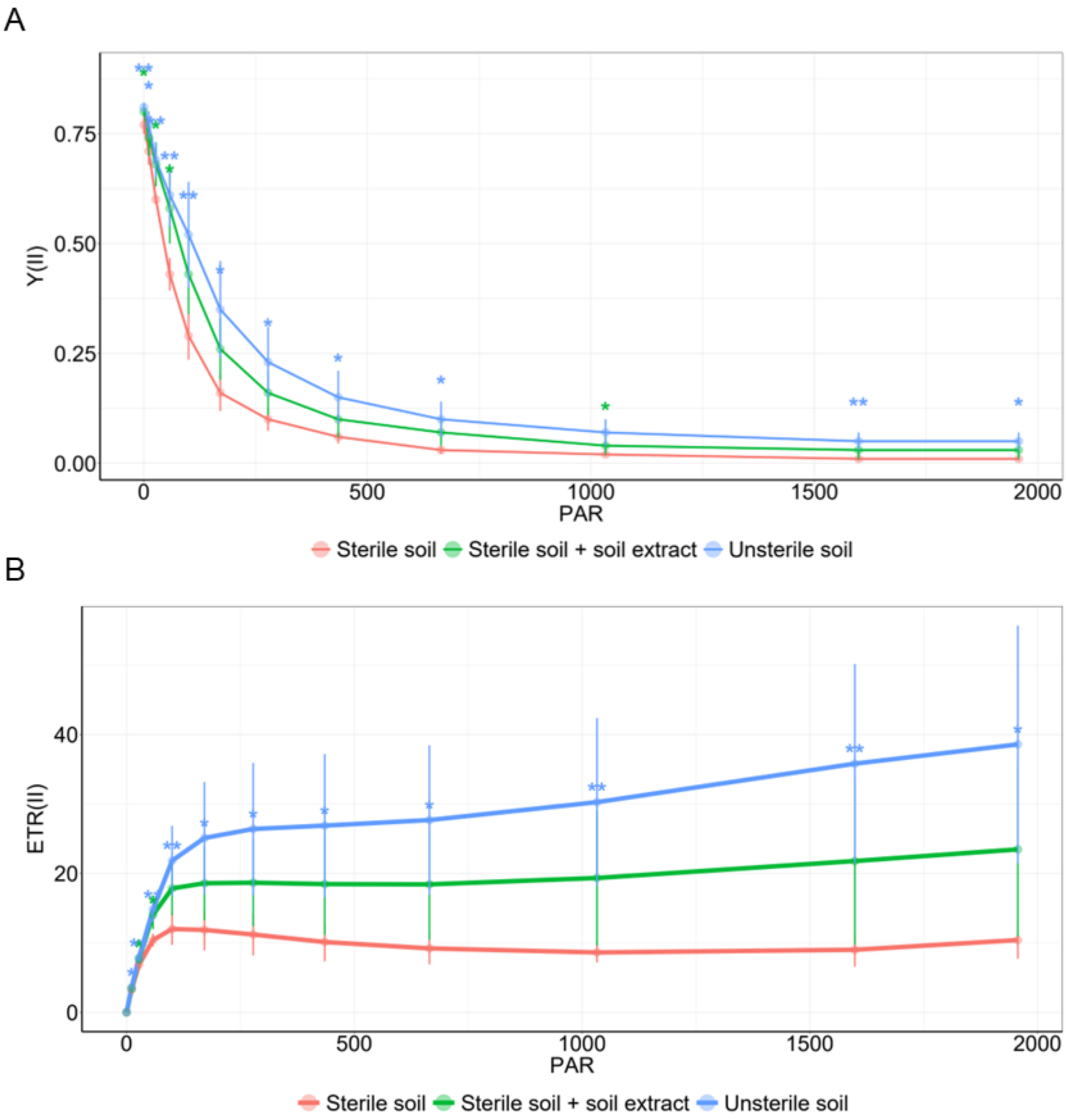

Supplementary fig. 5 Microorganisms enhance plant fitness. The effective photochemical quantum yield of photosystem II (YII) (A) and relative electron transfer (ETRII) (B) measured for *O. chalcidica* plants grown in sterilized serpentine soil (Sterile soil), sterilized serpentine soil supplemented with extract from serpentine soil (Sterile soil + soil extract) and in unsterile serpentine soil (Unsterile soil). The measurements were done for five plants per treatment (N=5). Stars indicate statistically significant differences (Dunnett's test, * - $P \leq 0.05$, ** - $P \leq 0.01$, *** - $P \leq 0.001$)

Electron transport in PSII was measured to assess plant performance across different microbiota treatments for *O. chalcidica*. Two key PSII parameters demonstrated improved photosystem II function, including effective quantum yield (YII) and relative electron transfer rates (ETRII), in *O. chalcidica* grown with natural serpentine microbiota or soil extract, indicating enhanced overall plant condition.

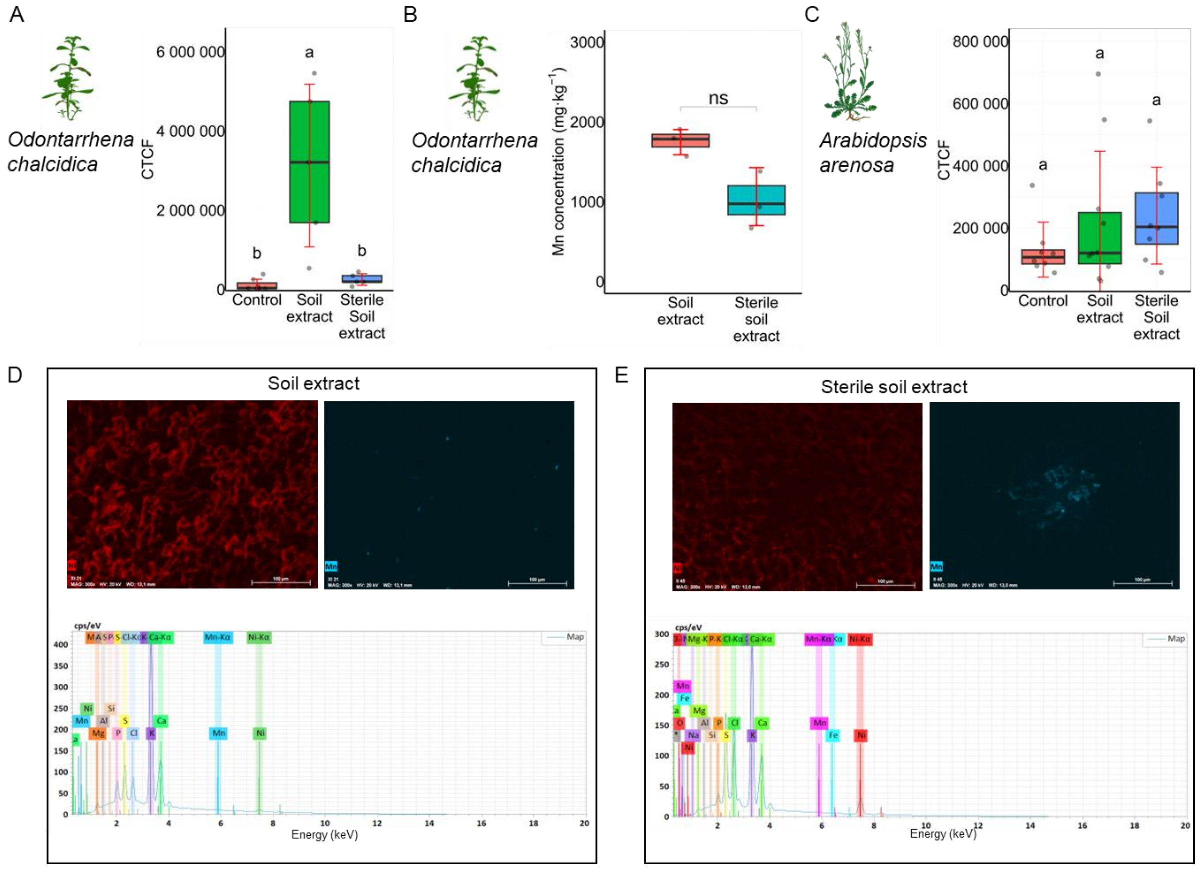

Supplementary fig. 6 Metals uptake in plants *in vitro.* (A) Corrected threshold cell fluorescence (CTCF) analysis of Ni staining with Newport Green™ DCF Diacetate in roots of *O. chalcidica* cultured *in vitro* in medium supplemented with 500 µM of Ni and 100 µM of Mn, treated with sterilized soil extract or with unsterilized serpentine soil extract. Plants without additional extract treatment served as a control (N=5). (B) Mn concentration in whole *O. chalcidica* plants cultured *in vitro* (N=3). (C) CTCF analysis of Ni staining in roots of *A. arenosa* cultured *in vitro* (N=3). Boxplots represent the median and interquartile range with standard deviation indicated in red. Stars indicate statistically significant differences (t-test, * - $P\leq0.05$, ** - $P\leq0.01$, *** - $P\leq0.001$) for pairwise comparisons and letters above bars denote significant differences between groups, as assessed by one-way ANOVA with Tukey's post hoc test for all multiple comparisons ($P\leq0.05$). (D, E) SEM-EDX images of the leaf surface of *O. chalcidica* plants inoculated with serpentine soil extract (D) and uninoculated controls (E), with corresponding elemental spectra shown below each image.

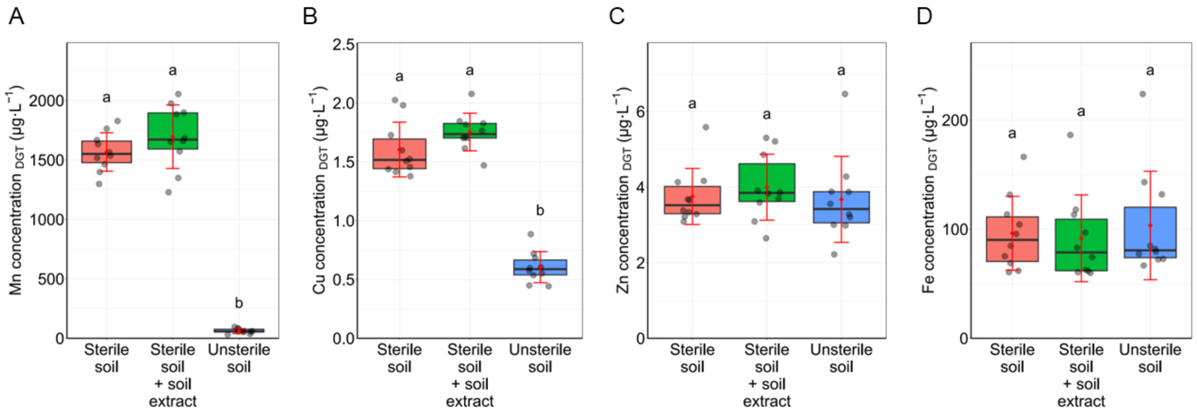

Supplementary fig. 7 Microbial effect on the concentration of DGT-mobile metals in serpentine soil. Impact of sterilization on the concentration of DGT-mobile metals in serpentine soil: concentration of Mn (A), Cu (B), Zn (C) and Fe (D) in sterilized serpentine soil (Sterile soil), sterilized serpentine soil supplemented with extract from serpentine soil (Sterile soil + soil extract) and in unsterile serpentine soil (Unsterile soil). Boxplots represent the median and interquartile range with standard deviation indicated in red. Letters above bars denote significant differences between groups, as assessed by one-way ANOVA with Tukey's post hoc test ($P \leq 0.05$) (N = 10).

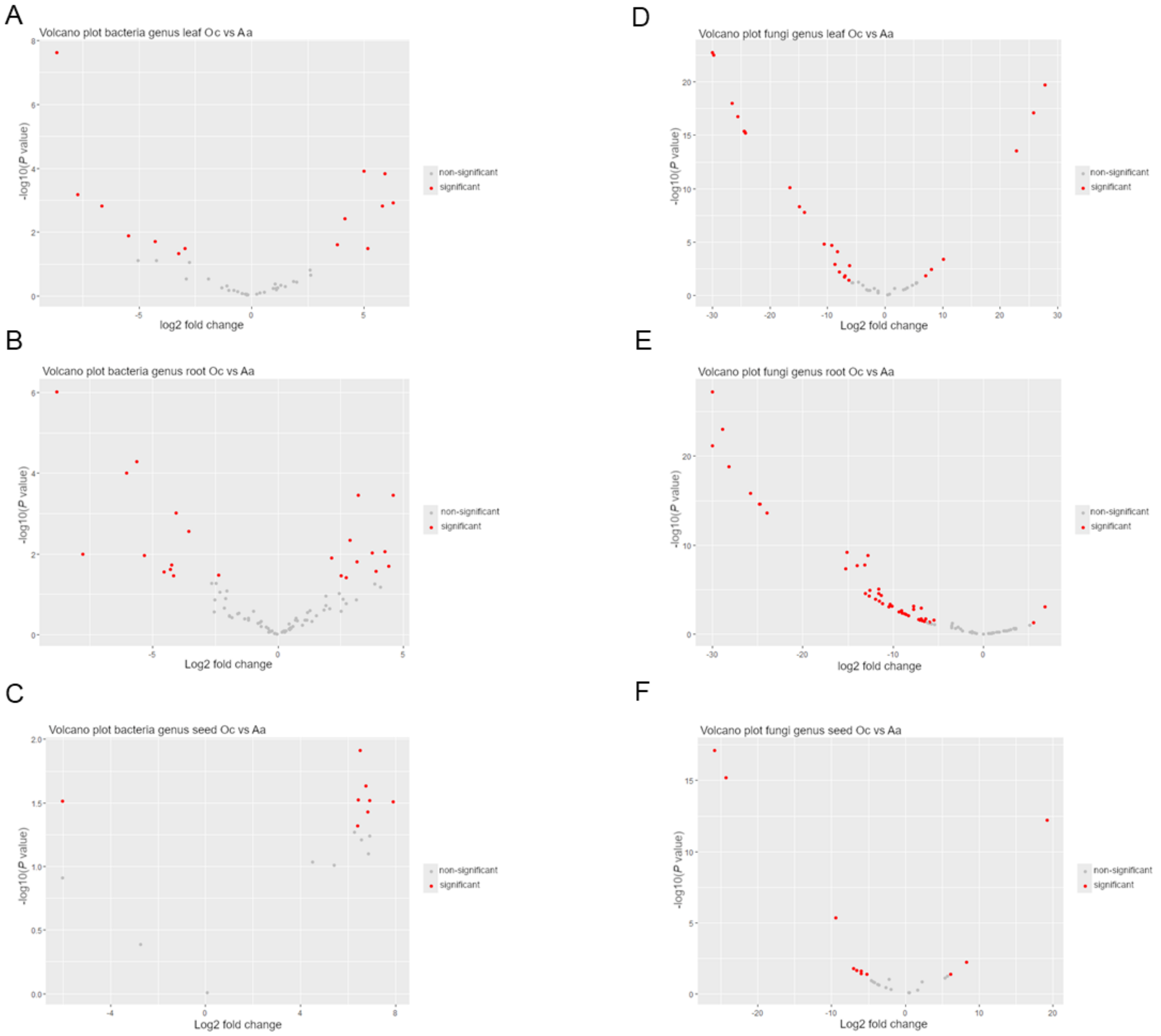

Supplementary fig. 8 The differentiation of fungal and bacterial taxa in *Odontarrhena chalcidica* and *Arabidopsis arenosa*. Volcano plots illustrating bacterial (A-C) and fungal (D-F) genera that were significantly enriched or depleted at genus level. Oc: *Odontarrhena chalcidica*, Aa: *Arabidopsis arenosa*.

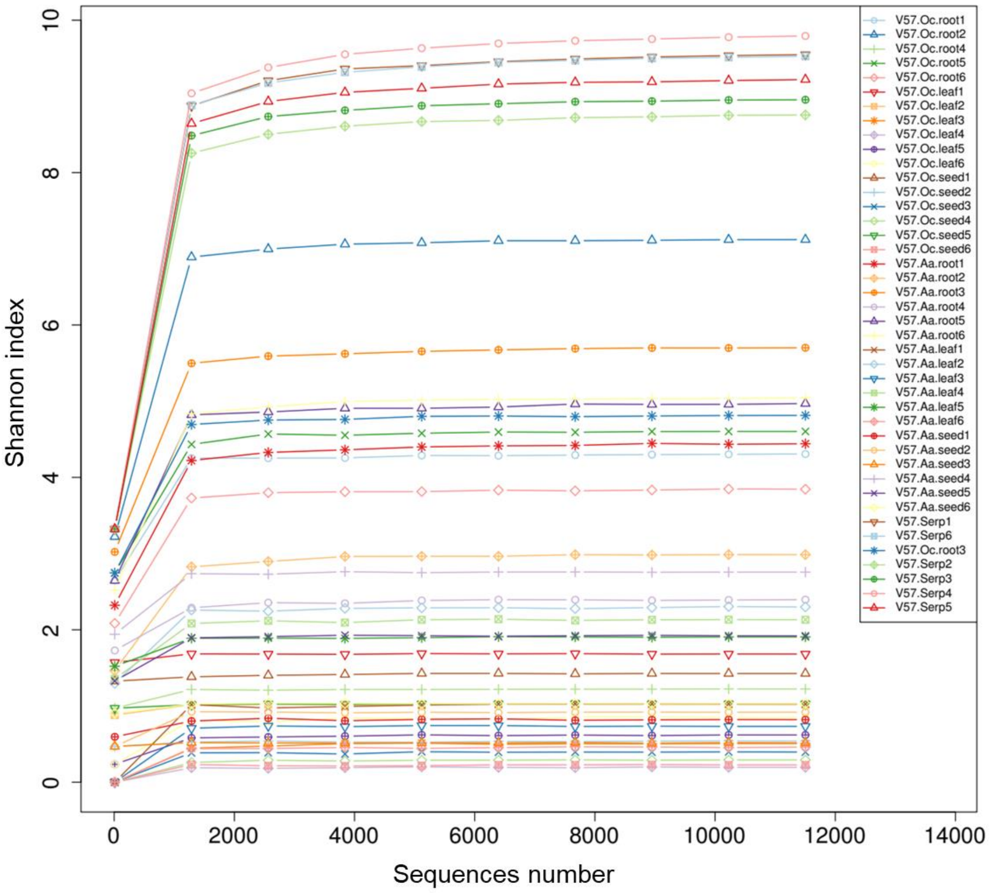

Supplementary fig. 9 Analysis of 16S rRNA gene sequencing depth. Refraction curve analysis with Shannon for each sample. Oc: *Odontarrhena chalcidica*, Aa: *Arabidopsis arenosa.*

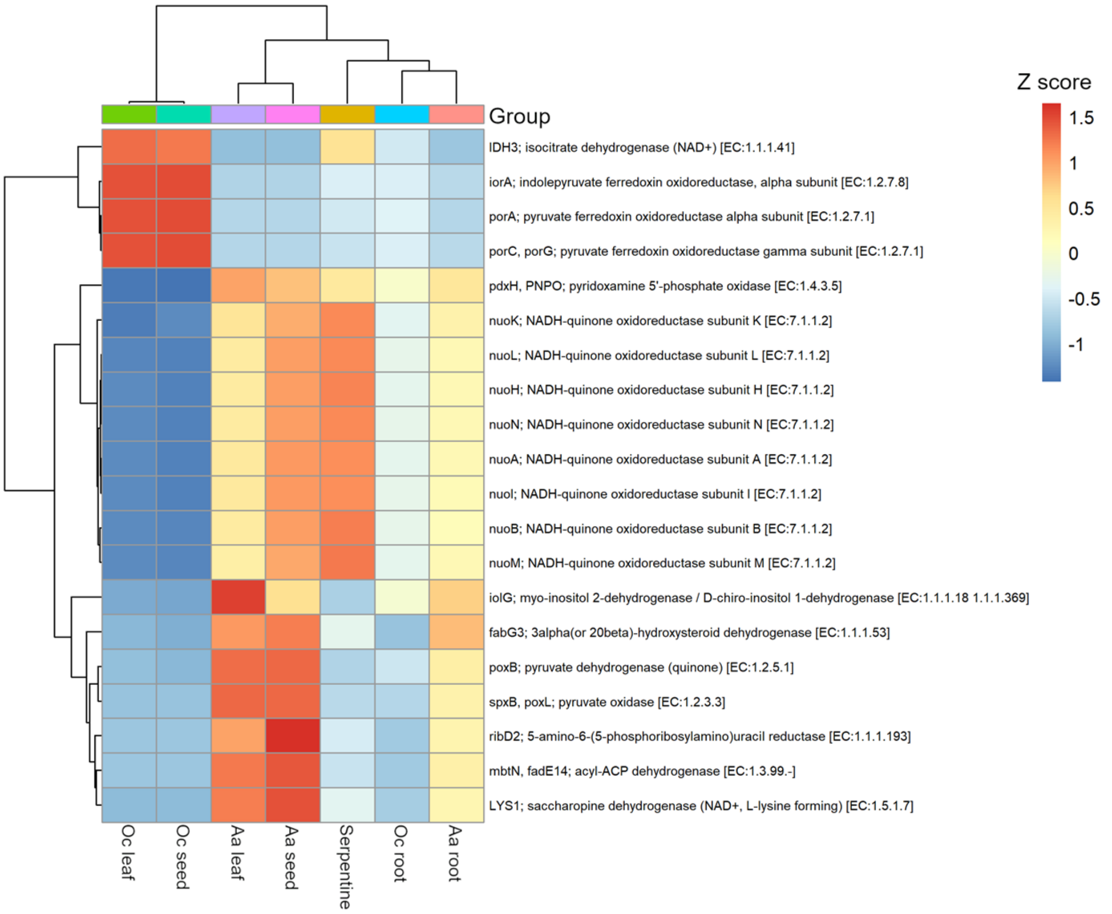

Supplementary fig. 10 Functional profiling of plant- and soil-associated bacterial microbiota. PICRUSt-constructed heatmap plot showing predictive functional KEGG profiles of bacterial microbiota associated with serpentine soil and *Odontarrhena chalcidica* (Oc) and *Arabidopsis arenosa* (Aa).

Predicted metagenomic functions were inferred from 16S rRNA gene-based community profiles using the PICRUSt algorithm and annotated against the Kyoto Encyclopedia of Genes and Genomes (KEGG) database. Comparative analysis of root, leaf, and seed-associated microbiota of *Odontarrhena calcidica* (Oc) and *Arabidopsis arenosa* (Aa), together with the surrounding serpentine soil, revealed clear host- and habitat-specific functional signatures (for details refer to Suppelementary table 10. Several genes associated with pyruvate metabolism and redox energy conversion, including *poxB* (pyruvate dehydrogenase [EC:1.2.5.1]), *spxb/poxL* (pyruvate oxidase [EC:1.2.3.3]), and *porA/C/G* (pyruvate:ferredoxin oxidoreductase [EC:1.2.7.1]), showed lower predicted abundances in the *O. chalcidica* microbiota, particularly in the root compartment, while being enriched in *A. arenosa* tissues. Likewise, multiple subunits of NADH–quinone oxidoreductase (*nuoA*–N; EC:7.1.1.2) were more abundant in *A. arenosa* and in the serpentine soil, suggesting a higher potential for oxidative phosphorylation and redox-driven energy metabolism in this microbiota. Heatmap clustering of predicted KEGG orthologs demonstrated a strong functional similarity between the serpentine soil and the root-associated microbiota of both plant species. This clustering pattern indicates that the serpentine soil exerts a major influence on the metabolic potential of root-associated microbial communities, likely through selective recruitment or environmental filtering of microbial taxa

with similar oxidative and dehydrogenase capacities. Despite this overall similarity, the *A. arenosa* root microbiota exhibited slightly higher predicted abundances of genes involved in energy metabolism (e.g., NADH–quinone oxidoreductase, pyruvate dehydrogenase), whereas the *O. chalcidica* root microbiota appeared functionally reduced, with a relative depletion of oxidative and cofactor-related pathways. In contrast, the *O. chalcidica* -associated microbiota showed lower predicted abundances of genes linked to cofactor and vitamin biosynthesis, including *iolG* (myo-inositol dehydrogenase), ribD2 (riboflavin biosynthesis), and *pdxH* (pyridoxamine 5′-phosphate oxidase), suggesting a more specialized or environmentally constrained functional repertoire. Enzymes such as *fabG3* (3α-hydroxysteroid dehydrogenase) and *LYS1* (saccharopine dehydrogenase) were enriched in *A. arenosa* pointing to a greater potential for amino acid and lipid metabolism in its associated microbiota.

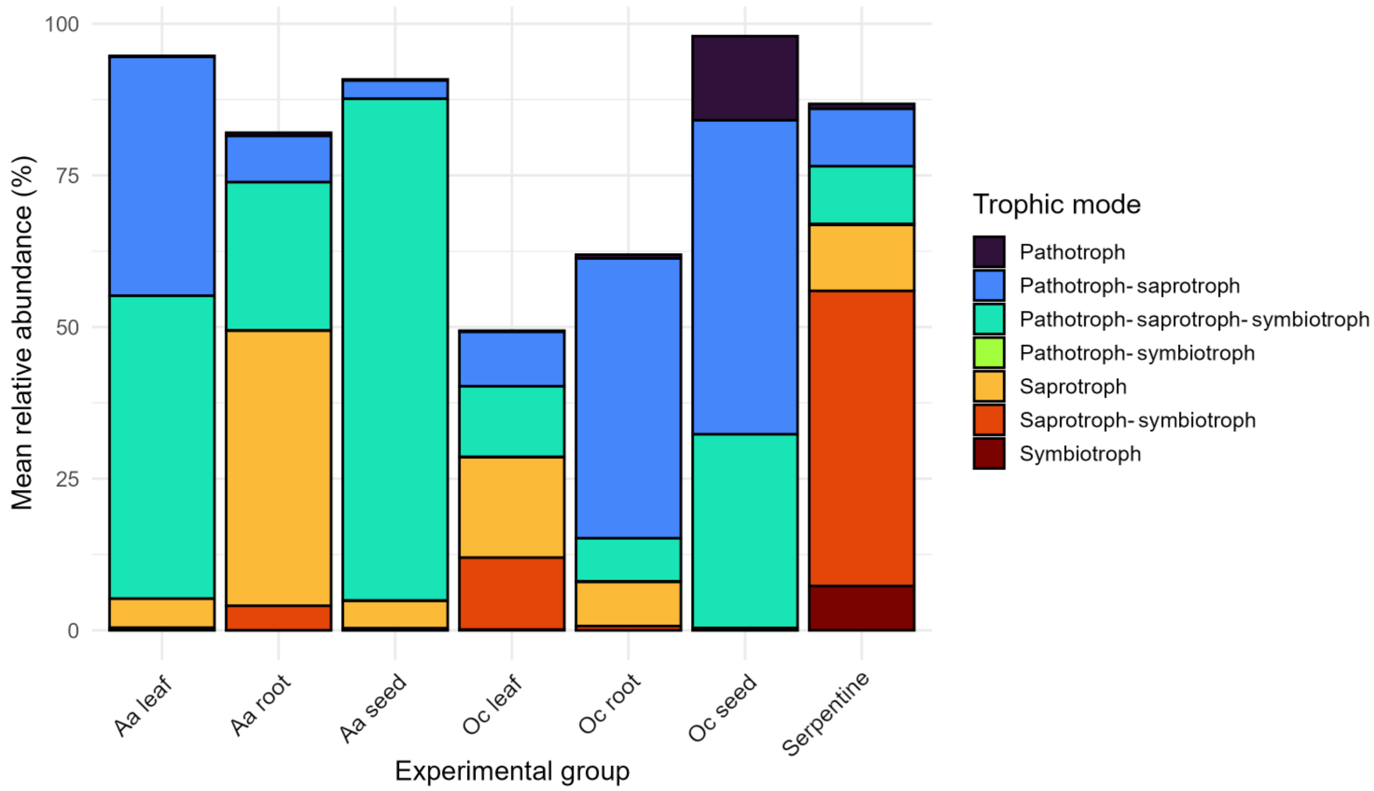

Supplementary fig. 11 Mean relative abundance of trophic modes across fungal communities associated with soil and plants. The stacked barplot showing the mean relative abundances of fungal trophic modes across sample groups, including leaves, roots, seeds of *A. arenosa* (Aa) and *O. calcidica* (Oc), and serpentine soil. Saprotrophs, symbiotrophs, and pathotrophs were the dominant functional categories, with some variation between plant organs and soil. Statistical differences between groups were assessed using Kruskal–Wallis and Dunn's post-hoc tests.

Fungal community functional composition was inferred using FUNGuild, which assigns ecological guilds to fungal taxa based on published functional annotations. The analysis included roots, leaves, and seeds of *A. arenosa* (Aa) and *O. chalcidica* (Oc), as well as the surrounding serpentine soil. Across all sample types, saprotroph-symbiotroph fungi were the most consistently detected guild, followed by pathotroph-symbiotroph and single-function guilds (saprotroph, symbiotroph, pathotroph) (for detailed results refer to Supplementary Table 11). Roots of both plant species were functionally more similar to each other and to serpentine soil than to leaves or seeds. *O. chalcidica* seed-associated fungi exhibited lower predicted abundances of symbiotrophic and pathotrophic guilds compared with *A. arenosa*, whereas serpentine soil harbored high relative abundances of saprotroph-symbiotroph fungi. Overall, these results suggest a strong overlap between root-associated and soil fungal functional potentials, highlighting the influence of host and edaphic environment on fungal guild distribution.

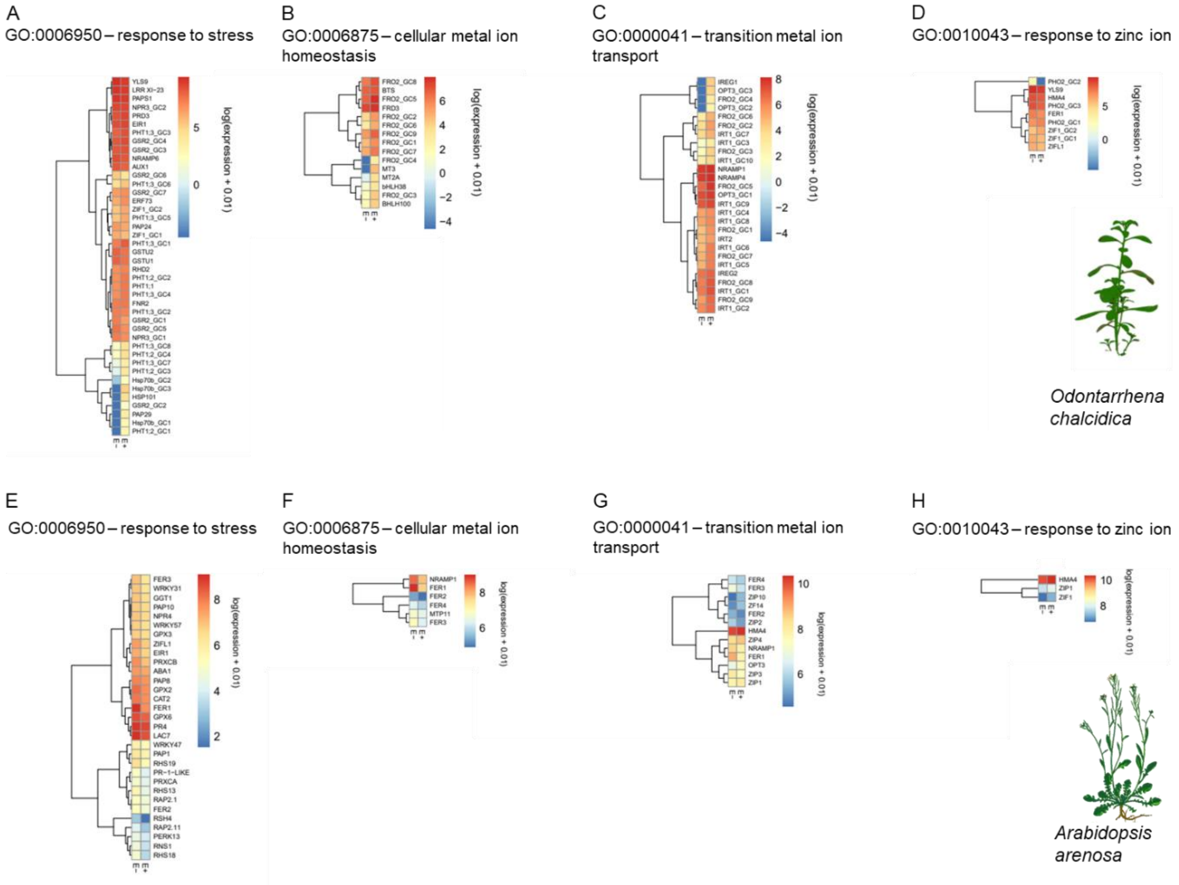

Supplementary fig. 12 The differentiation of fungal and bacterial taxa in *Odontarrhena chalcidica* and *Arabidopsis arenosa*. Heatmaps showing the expression of genes annotated to the GO categories "response to stress" (GO:0006950), "cellular metal ion homeostasis" (GO:0006875), "transition metal ion transport" (GO:0000041), and "response to zinc ion" (GO:0010043) in *O. chalcidica* (A–D) and *A. arenosa* (E–H), cultured *in vitro* for 7 days on medium supplemented with 500 µM Ni and 100 µM Mn and inoculated with serpentine soil extract (E+), and uninoculated (E-).
